# Awareness and acceptance of human papillomavirus vaccine in the Middle East: A systematic review, meta-analysis, and meta-regression of 159 studies

**DOI:** 10.1017/S0950268824001596

**Published:** 2024-12-10

**Authors:** Bugra Taygun Gulle, Pinar Kiran, Saadet Goksu Celik, Zeynep Sedef Varol, Neslisah Siyve, Ahmet Naci Emecen, Hilal Duzel

**Affiliations:** 1 Faculty of Medicine, Department of Public Health, Division of Epidemiology, Dokuz Eylül University, Izmir, Turkey; 2 Faculty of Medicine, Department of Public Health, Dokuz Eylül University, Izmir, Turkey; 3 Communicable Diseases Unit, Izmir Provincial Health Directorate, Izmir, Turkey; 4 Izmir Kemalpaşa District Health Directorate, Public Health Department, Izmir, Turkey

**Keywords:** human papillomavirus, vaccine acceptance, vaccine awareness, Middle East, meta-analysis, meta-regression

## Abstract

Cervical cancer, closely linked to human papillomavirus (HPV) infection, is a major global health concern. Our study aims to fill the gap in understanding HPV vaccine awareness and acceptance in the Middle East, where national immunization programs are often lacking and cultural perceptions hinder acceptance. This systematic review and meta-analysis adhered to Preferred Reporting Items for Systematic Reviews and Meta-Analyses guidelines. A comprehensive literature search across several databases was conducted on 5 September 2023. We included quantitative studies on HPV vaccine awareness and acceptance in Middle Eastern countries. Data extraction and quality assessment were conducted independently by multiple reviewers to ensure accuracy. Statistical analyses, including subgroup analyses, were performed using R to calculate pooled estimates, assess heterogeneity, and publication bias. We reviewed 159 articles from 15 Middle Eastern countries, focusing on 93,730 participants, predominantly female and healthcare workers. HPV vaccine awareness was found to be 41.7% (95% CI 37.4%–46.1%), with higher awareness among healthcare workers. The pooled acceptance rate was 45.6% (95% CI 41.3%–50.1%), with similar rates between healthcare and non-healthcare workers. Our study highlights the critical need for increased HPV vaccine awareness and acceptance in the Middle East, emphasizing the importance of integrating the vaccine into national immunization programs and addressing cultural and religious factors to improve public health outcomes.

## 1. Introduction

Cervical cancer stands as the fourth most prevalent cancer and a leading cause of cancer-related mortality among women globally. Disproportionate burdens of incidence and mortality characterize developing countries compared to their developed counterparts [1]. Persistent human papillomavirus (HPV) infection is identified as the primary cause of precancerous and cancerous cervical lesions, extending its association with cancers of various regions, including the head, neck, oropharynx, and anogenital areas [2–4].

The advent of the HPV vaccine in 2006 marked a significant milestone. The World Health Organization (WHO) underscores its importance, recommending its inclusion in all national immunization programs with the ambitious goal of reaching 90% of all girls by age 15 by 2030 [5]. However, the adoption of the HPV vaccine remains uneven globally. Notably, a substantial gap persists, especially in low-income countries, where less than 25% have integrated HPV vaccines into their vaccination schedules [2,6].

Vaccine hesitancy, characterized by reluctance or refusal to vaccinate despite the availability of vaccines, has been recognized as one of the top 10 threats to global health by the WHO [7]. Furthermore, the absence of HPV vaccination from national immunization programs may significantly influence the acceptance of HPV vaccine [8]. Even in settings where vaccines have been included in the national schedule, a notable level of distrust and hesitancy persists, especially when dealing with recently introduced vaccines such as the HPV vaccines [8,9]. The association of the HPV vaccine with a sexually transmitted disease, its administration to young girls, and its introduction in societies where such topics are often considered taboo, can impact vaccine acceptance [10]. Notably, in Muslim communities where premarital sexual activity is discouraged, there exist a perception that HPV vaccination is unnecessary, and some individuals even fear that it might encourage early sexual activity [8,11].

The Middle East, encompassing the southern and eastern shores of the Mediterranean Sea, including the Arabian Peninsula and Iran, presents a unique context [12]. In 14 out of 17 countries within the region, the HPV vaccine is not presented in the national immunization programs. Notably, 15 of these countries have a predominantly Muslim population, and six are categorized as having low or lower-middle income status [6,13]. Given these circumstances, the awareness and acceptance of HPV vaccination in the Middle East emerge as pivotal considerations.

Despite systematic reviews and meta-analyses examining HPV vaccine awareness and acceptance globally, a conspicuous gap persists in the literature concerning the Middle East [14–18]. To address this knowledge void, our study aims to provide a comprehensive and region-specific understanding of the status of HPV vaccine awareness and acceptance in the Middle East.

## 2. Materials and methods

### 2.1 Protocol and registration

The protocol of this systematic review and meta-analysis was registered with the International Prospective Register of Systematic Reviews (PROSPERO) on 2 September 2023 (CRD42023456653) and to our knowledge, no similar studies have been registered. This study was reported in accordance with the Preferred Reporting Items for Systematic Reviews and Meta-Analyses (PRISMA) guideline [19].

### 2.2 Search strategy

A comprehensive literature search was conducted in MEDLINE (via PubMed), Web of Science (WoS), TürkMedline (National Health Sciences – Periodicals Database), TRDIZIN (TUBITAK-ULAKBIM). The electronic databases were searched on 5 September 2023. The search was conducted by integrating the words ‘acceptance’, ‘hesitancy’, ‘awareness’, ‘knowledge’, ‘attitude’, ‘willingness’, ‘Middle East’, ‘HPV’, ‘HPV vaccine’ using the ‘and’ and ‘or’ boolean operators. Further details on the search strategy are provided in Supplementary Materials.

### 2.3 Selection criteria

Articles that met the following criteria were included in our study:

- Articles reported quantitative results related to HPV vaccine awareness or acceptance.
- Cross-sectional studies.
- Studies conducted in Middle East Countries (Iran, Turkey, Iraq, Saudi Arabia, Egypt, Yemen, Syria, Jordan, United Arab Emirates [UAE], Israel, Palestine, Lebanon, Oman, Kuwait, Qatar, Bahrain, Cyprus).
- Articles written in English or Turkish.
- Peer reviewed, original, and published articles.
- Articles with full-text available.

The questions ‘Have you heard/know about the HPV vaccine?’ were used to measure HPV vaccine awareness, while the questions ‘Are you willing/do you want/are you accepting of getting the HPV vaccine?’ were used to measure HPV vaccine acceptance.

In this study, if 90% or more of the study population consisted of healthcare workers or students (HCWS), the study was classified as being conducted among HCWS. If the percentage of HCWS was below 10%, the study was classified as being conducted among non-HCWS.

### 2.4 Selection process and data extraction

After conducting the initial article search, duplicated articles were removed. Subsequently, two authors (N.Ş. and H.D.) independently and blindly screened the titles and abstracts of the studies. The full text of all records that passed the title and abstract screening were retrieved and reviewed by three pairs of independent reviewers to confirm final eligibility (B.T.G., P.K., A.N.E., Z.S.V., N.Ş., S.G.Ç.). Any discrepancies in full-text review were resolved through discussion involving three authors (B.T.G, P.K, H.D.).

Data extraction form included the following items: author, title, year of publication, study period, sample size, participant characteristics, HPV vaccine awareness, and HPV vaccine acceptance were extracted using Microsoft Excel.

### 2.5 Quality assessment

The quality assessment of the chosen studies was conducted using the ‘Joanna Briggs Institute (JBI) Checklist for Prevalence Studies’. This tool assesses different aspects of study methodology, such as sampling strategies, data collection methods, and analytical techniques. Each of the nine checklist items was assigned a score of ‘0’ or ‘1’, resulting in a total score ranging from 0 to 9. Higher scores indicate a lower risk of bias [20].

### 2.6 Statistical analysis

Statistical analyses were performed using R version 4.3.2 with the ‘meta’ package. Pooled estimates of HPV vaccine awareness and HPV vaccine acceptance (for oneself and/or their child) were calculated separately and are presented with a 95% confidence interval (CI). A random effects model was used to estimate the pooled prevalence. Heterogeneity across studies was assessed using the *p*-value of the chi squared test and *I* ^2^ statistics. Statistically significant heterogeneity was considered when the *I* ^2^ value exceeded 40% or when the *p*-value of the chi-square test was below 0.1 [21]. Subgroup analyses were conducted based on countries, healthcare populations, and study enrolment time. Publication bias was assessed and adjusted through the implementation of Egger’s regression test and the ‘trim and fill’ method [22]. For the meta-regression, we employed the restricted maximum-likelihood method to investigate the influence of study-level characteristics (percentage of healthcare workers (HCWs), study year, number of participants, percentage of females in the studies, and the total bias score) on the variation in HPV vaccine awareness and acceptance.

## 3. Results

### 3.1 Study selection

**Figure 1.**
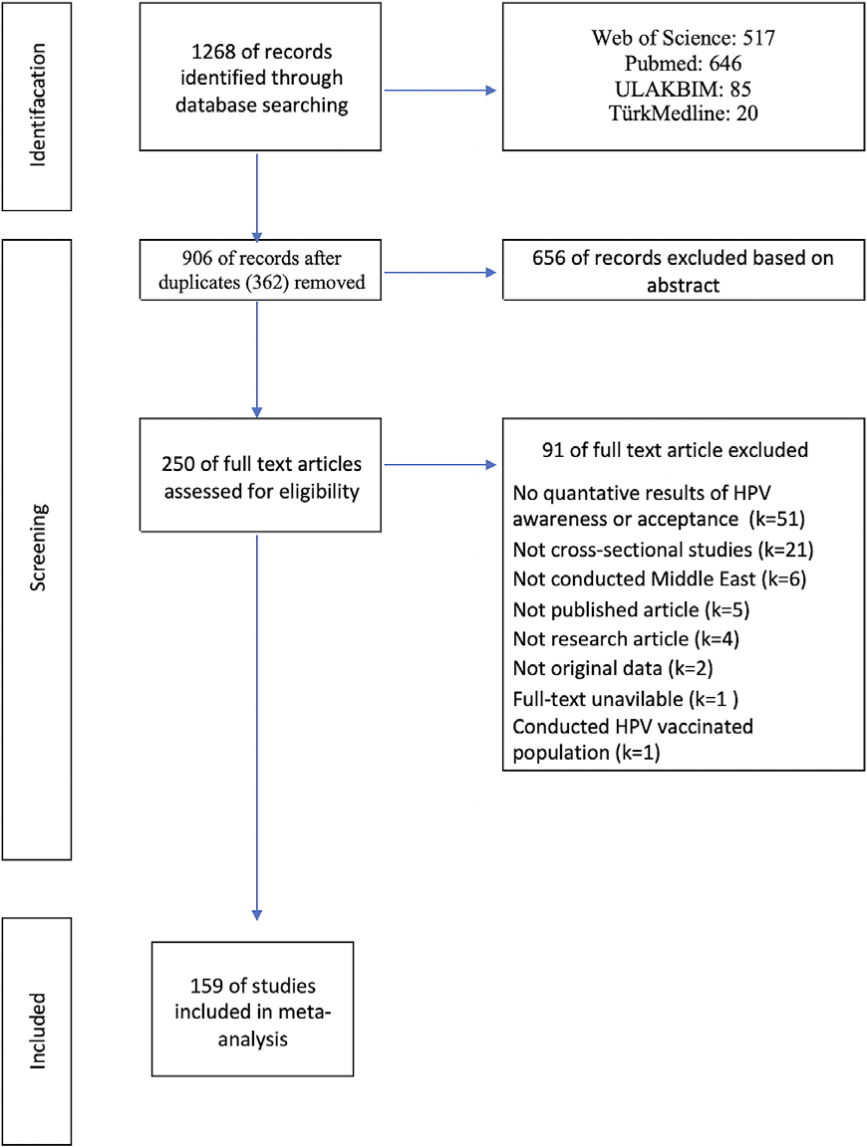
Flow diagram of article selection.

After conducting a thorough literature review, a total of 1,268 articles were identified. Following the removal of 362 duplicate articles, 656 studies were excluded based on the examination of titles and abstracts for not meeting the specified inclusion criteria. The full texts of the remaining 250 articles were reviewed, leading to the exclusion of 91 articles that did not meet the inclusion criteria, as detailed in Supplementary Materials (Table 1). Ultimately, 159 articles were included in the study, with selection process illustrated in Figure 1, in accordance with PRISMA guidelines [19].

**Table 1.** HPV vaccine awareness and acceptance in the Middle East countries

Country^a^	HPV vaccine awareness			HPV vaccine acceptance		
	Number of studies	Pooled prevalence (%) (95% CI)	*I* ^2^ (%)	Number of studies	Pooled prevalence (%) (95% CI)	*I* ^2^ (%)
*Turkey*	*74*	44.9 (39.2–50.6)	99.7	57	39.0 (33.7–44.3)	99.4
Healthcare-related^b^	26	59.8 (49.8–69.8)	99.5	23	42.1 (34.1–50.1)	98.9
Not healthcare-related^b^	37	33.4 (26.6–40.3)	99.6	24	37.0 (28.2–45.8)	99.6
*Saudi Arabia*	*27*	31.4 (24.0–38.8)	99.0	14	51.1 (43.5–58.8)	98.1
Healthcare-related	7	53.7 (40.1–67.2)	98.4	3	44.7 (40.2–59.3)	81.2
Not healthcare-related	17	20.4 (15.2–25.6)	97.7	10	53.7 (43.4–64.0)	98.4
*UAE^c^*	*6*	42.8 (20.2–65.5)	99.9	5	57.1 (40.8–73.4)	98.7
Healthcare-related	2	55.1 (0–100)	100	1	74.4 (66.8.–82.1)	
Not healthcare-related	4	36.7 (32.9–40.4)	65	4	52.9 (34.8–70.9)	99.0
*Cyprus*	*3*	54.5 (17.5–81.6)	99.1	2	57.1 (5.4–100)	99.5
Healthcare-related	*1*	81.7 (76.3–87.1)	–	1	83.5 (78.4–88.6)	–
Not healthcare-related	*2*	40.5 (31.9–49.1)	83.2	1	30.7 (25.3–36.1)	–
*Jordan*	*3*	39.3 (23.8–54.8)	98.8	2	58.5 (26.2–90.8)	99.6
Healthcare-related	*1*	48.7 (45.3–52.1)	–	1	75 (72.1–77.9)	–
Not healthcare-related	2	34.6 (13.2–56.1)	98.2	1	42 (39.2–44.8)	–
*Lebanon*	*3*	54.7 (34.3–75.1)	98.9	1	52.0 (47.3–56.6)	–
Healthcare-related	–	–	–	–	–	–
Not healthcare-related	*2*	50.4 (18.6–82.3)	99.4	1	52.0 (47.3–56.6)	–
*Bahrein*	2	17.5 (0–44.5)	99.3	3	83.2 (74.3–92.0)	95.6
Healthcare-related	–	–	–	–	–	–
Not healthcare-related	2	17.5 (0–44.5)	99.3	2	86.7 (77.4–96.0)	93.0
*Iraq*	2	43.8 (1.3–86.3)	99.7	1	45.8 (40.9–50.7)	–
Healthcare-related	1	65.5 (62.8–68.2)	–	–	–	–
Not healthcare-related	1	22.1 (18.7–25.5)	–	1	45.8 (40.9–50.7)	
*Oman*	2	15.5 (4.8–26.2)	97.3	2	65.9 (50.4–81.4)	98.3
Healthcare-related	–	–	–	–	–	–
Not healthcare-related	2	15.5 (4.8–26.2)	97.3	2	65.9 (50.4–81.4)	98.3
*Egypt*	1	10.8 (5.0–16.6)	–	–	–	–
Healthcare-related	1	10.8 (5.0–16.6)	–	–	–	–
Not healthcare-related	–	–	–	–	–	–
*Qatar*	1	18.6 (14.8–22.4)	–	1	35.5 (31.6–39.4)	–
Healthcare-related	–	–	–	–	–	–
Not healthcare-related	1	18.6 (14.8–22.4)	–	1	35.5 (31.6–39.4)	–
Palestine	1	0.65 (0.46–0.84)	–	–	–	–
Healthcare-related	–	0.65 (0.46–0.84)	–	–	–	–
Not healthcare-related	1	–	–	–	–	–
Israel	1	84 (79.9–88.1)	–	2	63.4 (58.8–68.1)	0
Healthcare-related	–	–	–	–	–	–
Not healthcare-related	1	84 (79.9–88.1)	–	2	63.4 (58.8–68.1)	0
Iran	1	60.1 (54.5–65.7)	–	2	51.6 (31.1–72.1)	96.6
Healthcare-related	1	60.1 (54.5–65.7)	–	2	51.6 (31.1–72.1)	96.6
Not healthcare-related	–	–	–	–	–	–
*Kuwait*	1	43.5 (39.6–54.8)	–	1	38.6 (34.7–42.5)	–
Healthcare-related	–	–	–	–	–	–
Not healthcare-related	–	–	–	–	–	–
*Total*	*123*	41.7 (37.4–46.1)	99.8	90	45.6 (41.3–50.1)	99.4
Healthcare-related	38	59.9 (52.2–67.5)	98.1	31	46.5 (39.4–53.5)	99.0
Not healthcare-related	69	30.4 (25.7–35.1)	99.7	46	46.7 (40.2–53.1)	99.6

Non-healthcare-related: Refers to the studies where 10% or less of the study population consisted of healthcare workers or students.

The total number of studies may differ from the sum of these two categories, as it also includes studies where the proportion of healthcare workers or students is between 10% and 90%.

a The results of two studies conducted in multiple countries were included in the subgroup analysis based on countries, with outcomes separated according to each country.

b Healthcare-related: Refers to the studies where at least 90% of the study population consisted of healthcare workers or students.

c United Arab Emirates.

### 3.2 Characteristics of the included studies

This systematic review and meta-analysis included a total of 159 articles conducted in 15 different countries, spanning the years 2007–2023, and involving a combined sample of 93,730 individuals. Notably, in these studies, 81.4% of participants were female, and within the participant pool, 25.4% were HCWS. Out of these studies, 123 were included in the analyses for HPV vaccine awareness, 90 for HPV vaccine acceptance, 15 for child-specific HPV vaccine acceptance, 41 for HPV vaccine acceptance in female children, and 16 for HPV vaccine acceptance in male children. Then, 95 of the included studies were conducted in Turkey, while 32 were carried out in Saudi Arabia. Additionally, two studies were conducted in multiple countries [21,22]. Among the included studies, 52 were conducted exclusively among HCWS. The descriptive characteristics of the included studies are summarized in Supplementary Materials (Table 2) [23–178].

**Table 2.** Meta-regression of HPV vaccine awareness

Variable	β	se	*p*	95% Confidence interval	
				Lower	Upper
Number of participants (per100 participants)	−0.5519	0.2670	**0.0387**	−1.0572	−0.0287
Study enrolment time (year)^a^	−0.0788	0.4773	0.8680	−1.0144	0.8567
Health-related population (%)	0.2605	0.0427	**<0.001**	0.1768	0.3441
Female participant (%)^b^	−0.0836	0.0808	0.3011	−0.2419	0.0748
Total bias points	−2.0735	1.4441	0.1510	−4.9038	0.7568
Intercept	213.3286	964.7110	0.825		
tau^2^ = 404.1704, I^2^ = %99.35	*R* ^2^ = 32.07				

a If the study spanned multiple years, the initiation year of the study was used. In cases where the study year was not explicitly stated, the study enrolment year was considered, which was 1 year prior of the publication year of the article.

b For one study in which the percentage of female participants was not provided, it was assumed to be 50%.

### 3.3 HPV vaccine awareness

#### 3.3.1 Characteristics of the included studies

A total of 123 studies, were included in the analyses for HPV vaccine awareness. Then, 74 studies were conducted in Turkey, 27 in Saudi Arabia, and six in UAE. Of the total 73,116 individuals included in the studies, 84.25% are female, and 22.8% are HCWS. Of these 123 studies, 62 were conducted after 2017, while 61 studies were conducted before 2018.

#### 3.3.2 Pooled prevalence and subgroup analysis

The pooled prevalence of HPV vaccine awareness from 123 studies was found to be 41.7% (95% CI 37.4–46.1, *I* ^2^ = 99.8%). Among studies conducted on HCWS (*n* = 38) the pooled prevalence of HPV vaccine awareness was 59.9% (95% CI 52.2–67.5, *I* ^2^ = 99.4%), while in studies conducted on individuals not HCWS (*n* = 73), it was 30.4% (95% CI 25.7–35.1, *I* ^2^ = 99.7%). A total of 38 studies were conducted among HCWS with 15 studies specifically focused on those in HCWs, and 22 studies targeting healthcare students (HCS) (one study was not included in both groups because it was conducted in both populations). Subgroup analysis showed that HPV vaccine awareness among HCWs was 77.4% (95% CI 67.4–87.4, *I* ^2^ = 98.3%), while awareness among healthcare students (HCS) was found to be 46.3% (95% CI 39.2–53.4, *I* ^2^ = 98.8%). In studies conducted in 2018 and later, HPV vaccine awareness was found to be 40.9% (95% CI 35–46.9, *I* ^2^ = 99.8%), while studies conducted in 2017 and earlier reported an awareness level of 42.6% (95% CI 36.2–48.9, *I* ^2^ = 99.7%).

**Figure 2.**
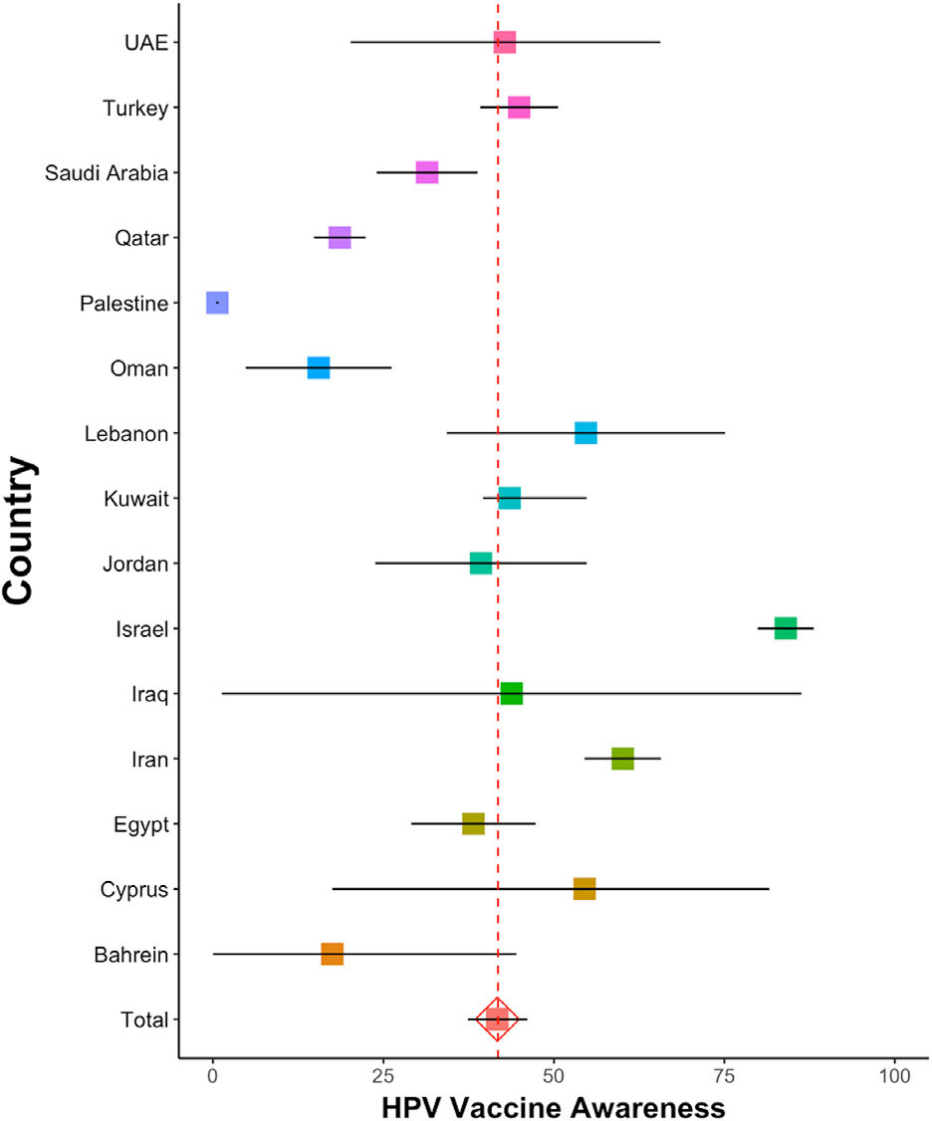
HPV vaccine awareness by country.

In a singular study involving 7,058 women in Palestine, which stands as the sole research conducted in Palestine, only 46 individuals (0.65%) were reported to be aware of the HPV vaccine, representing the study with the lowest HPV vaccine awareness among non-HCWS [134]. Conversely, the study exhibiting the highest HPV vaccine awareness among non-HCWS conducted in Israel, encompassing the mothers of 313 8th-grade children. In this study, HPV vaccine awareness was found to be 84% [90]. In a multinational study, particularly within the section carried out in the UAE, it was noted that merely 13.3% of 90 dental students were aware of the presence of the HPV vaccine, marking the lowest percentage among HCWS [150]. Additionally, numerous studies consistently report HPV vaccine awareness among HCWs to surpass 90% [55,60,81,154,165,174]. Supplementary Materials (Figure 1) provides forest plots illustrating the results of HPV vaccine awareness, while Table 1 and Figure 2 summarize the pooled prevalences, categorized by country.

### 3.4 HPV vaccine acceptance

#### 3.4.1 Characteristics of the included studies

Analysis for HPV vaccine acceptance included a total of 90 studies involving 50,696 participants. Of these studies, 57 were conducted in Turkey, 14 in Saudi Arabia, and 5 in the UAE. Of the participants, 79.8% were female, and 31% were HCWS. It is observed that 38 studies (42.2%) were conducted after 2017, while 52 studies (57.8%) were conducted before 2018.

The pooled prevalence of HPV vaccine acceptance was found to be 45.6% (95% CI 41.3–50.1, *I* ^2^ = 99.4%). Among these studies, the prevalence among HCWS was 46.5 (95% CI 39.4–53.5, *I* ^2^ = 99.0%), while in the non-HCWS group, the prevalence was 46.7 (95% CI 40.4–53.6, *I* ^2^ = 99.6%). In studies conducted after 2017, HPV vaccine acceptance was found to be 48.0% (95% CI 41.6–54.4, *I* ^2^ = 99.4%), while in those conducted before 2018, the acceptance was 44.0% (95% CI 38.0–50.0, *I* ^2^ = 99.4%).

In a 2013 study conducted in Turkey with 501 women aged 13–18, only 6.3% expressed willingness to receive the HPV vaccine [45]. Another study conducted in Turkey in 2012, involving 718 university students, reported HPV vaccine acceptance at 8.8% [41]. In a 2011 Bahrain study involving 571 women who provided cervical samples, a notable 91.3% expressed interest in receiving vaccination if it was accessible [74]. Similarly, a 2015 study in Cyprus with 200 female HCWs reported a notable 83.5% acceptance of the HPV vaccine [47].

The pooled prevalence of HPV vaccine acceptance for children in 15 studies was 52.8% (95% CI 40.4–65.2, *I* ^2^ = 99.7%). Specifically focusing on the acceptance of the HPV vaccine for girls in 41 studies resulted in a pooled prevalence of 55.6% (95% CI 48.7–62.4, *I* ^2^ = 99.4%). Similarly, for boys, the pooled prevalence of HPV vaccine acceptance, investigated in 16 studies, was 47.4% (95% CI 37.4–57.5 *I* ^2^ = 99.2%).

**Figure 3.**
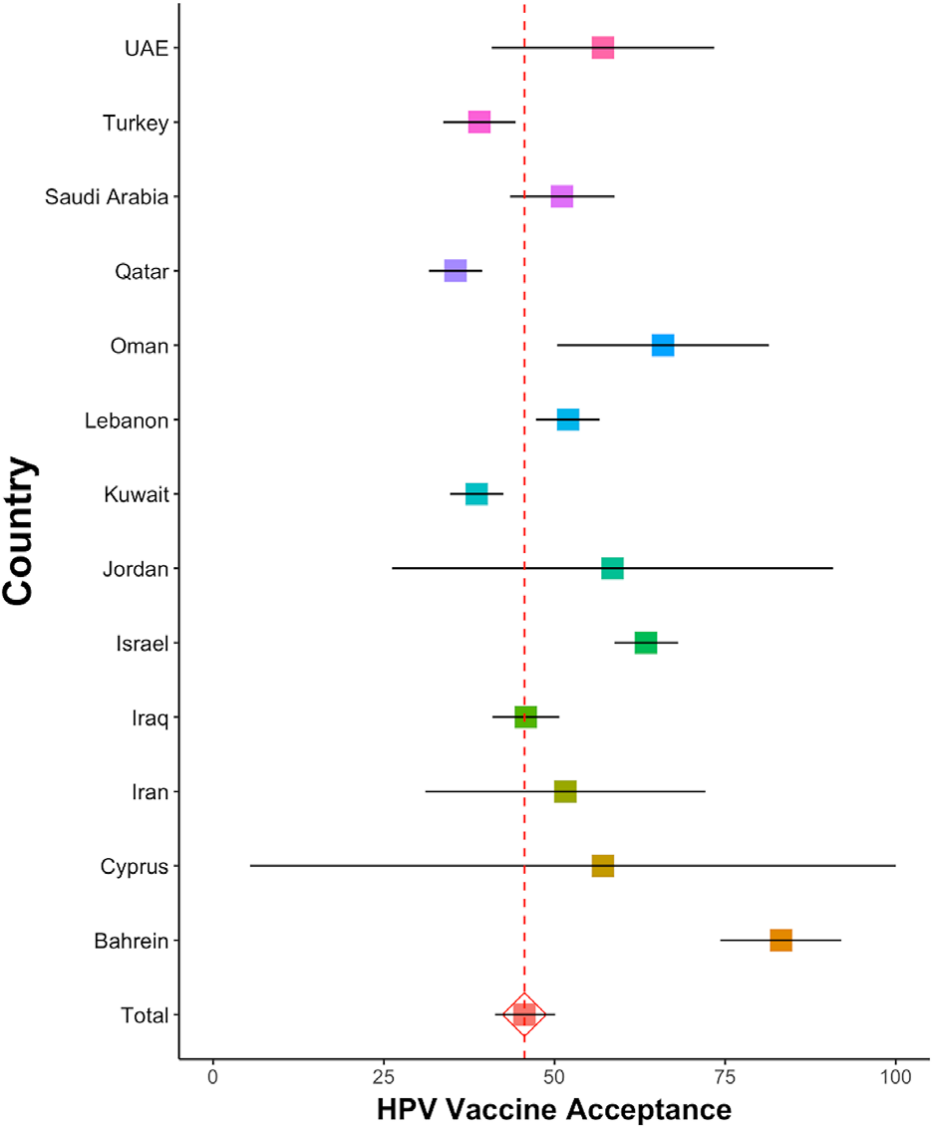
HPV vaccine acceptance by country.

Forest plots for the results of HPV vaccine acceptance provided in Supplementary Material (Figure 2). The pooled prevalences of HPV vaccine acceptance, grouped by country and HCWS, are summarized in Table 1 and Figure 3.

### 3.5 Bias analysis

The studies included in the research were evaluated using the JBI Checklist for Prevalence Study, and detailed assessment results are provided in Supplementary Materials. Upon reviewing these studies, limitations were identified in terms of identifying the target groups, selecting an appropriate sampling method, reporting the access rates of selected individuals, and providing data for unreachable groups.

Due to substantial heterogeneity and a large number of studies, funnel plots were not generated for HPV vaccine awareness and acceptance. The Egger’s tests revealed an asymmetric distribution (*p* < 0.001). The trim-and-fill method was employed to assess and address potential publication bias, and it was found that inclusion of additional studies was not required for the analysis of both outcomes. The pooled estimate results after applying the trim-and-fill method for HPV vaccine awareness were 41.7% (95% CI 37.4%–46.1%), and for HPV vaccine acceptance, the pooled estimate remained 45.6% (95% CI 41.3%–50.1%).

### 3.6 Meta-regression

The meta-regression for HPV vaccine awareness included variables such as the percentage of HCWs, study year, number of participants, percentage of females in the studies, and the total bias score (tau^2^ = 404.17, *I* ^2^ = 99.35%, *R* ^2^ = 32.07). A statistically significant relationship was observed between the number of participants (per 100 participants) (β = −0.55, 95% CI −1.05 to −0.03, *p* = 0.039) and the percentage of HCWs (β = 0.26, 95% CI 0.18–0.34, *p* < 0.001) in the study with HPV vaccine awareness. The detailed results of the meta-regression are provided in Table 2. When we conducted a meta-regression analysis for HPV vaccine acceptance using the same variables, it was observed that no significant or explanatory model emerged.

## 4. Discussion

In our meta-analysis, awareness of the HPV vaccine in the Middle East was found to be 41.7%, while it stood at 30.4% among non-HCWS. A 2016 meta-analysis of 43 studies in China reported an HPV vaccine awareness level of 15.95%. Subsequently, a 2021 meta-analysis comprising 36 studies focused on college students in China revealed an HPV vaccine awareness of 40.27%. A more recent meta-analysis in 2023, which included 15 studies on high school students in China, reported an awareness of 28.11% [14,179,180]. Additionally, a 2023 meta-analysis covering eight studies in Ethiopia found a good level of knowledge of the HPV vaccine at 55.12% [181]. A systematic review of studies conducted in Sub-Saharan African countries included 16 studies; only one demonstrated a moderately high level of knowledge, with the rest reporting low to no knowledge [182].

Awareness and knowledge of the HPV vaccine tend to be lower in regions such as China, Sub-Saharan Africa, and the Middle East. It is observed that HPV vaccine awareness is higher in countries where the vaccine is included in immunization programs and Western culture is predominant. For instance, in 2013, awareness of the HPV vaccine was between 67.1% and 71.3% in the United States, United Kingdom, and Australia [183]. A 2022 Swiss study found 83% awareness among participants aged 15–26 years, and a 2013–2014 US study reported a 66% awareness of HPV vaccination [184,185]. The Health Information National Trends Survey indicated that from 2008 to 2018, US awareness fluctuated between 60% and 65% [186]. Regional differences in vaccine awareness could be attributed to cultural and religious factors, as well as the priority given to the HPV vaccine within health systems.

Significant differences in HPV vaccine awareness were observed in our study between countries in the Middle East. In our meta-analysis, the largest number of studies was conducted in Turkey, Saudi Arabia, and the UAE, with reported awareness levels ranging from 31.4% to 44.9%. In the non-HCWS population, awareness ranged from 20.4% to 36.7%. These findings are consistent with those from countries where the HPV vaccine is not part of the official immunization program. Notably, while the UAE has included the HPV vaccine in its immunization schedule since 2018, most of the studies analysed were conducted prior to this date [6].

The results from other countries in our study should be interpreted with caution due to the smaller number of studies. Among the surveyed countries, Palestine is notable for its particularly low awareness of the HPV vaccine. Despite the availability of only one study, this research, which encompassed 7,058 women, revealed that a mere 46 participants (0.65%) were aware of the HPV vaccine [134]. Competing health concerns in Palestine may have resulted in the HPV vaccine receiving less attention.

Israel is distinguished by its unique cultural and religious composition, as well as the longstanding inclusion of the HPV vaccine in its immunization program. This long-term usage may contribute to the perception that vaccine awareness is not a significant issue, reflected by the inclusion of only one study in our meta-analysis. Conducted in 2017, this study reported an HPV vaccine awareness of 84%, which aligns with the higher rates observed in Western countries where the vaccine is routinely administered [90].

According to the results of the meta-regression, aside from the percentage of HCWs and the number of participants included in the study, no other examined variables showed a statistically significant relationship with the awareness of the HPV vaccine. The subgroup analysis also found that studies conducted among HCWs showed higher HPV vaccine awareness compared to those not involving HCWs. An increase in the number of participants in the study was statistically associated with a decrease in HPV vaccine awareness. However, as seen in the meta-regression results, for every increase of 100 participants, there is a 0.55% decrease in HPV vaccine awareness. While the relationship is found to be statistically significant, the size of the association is deemed to be negligibly small. Previous research suggests a positive relationship between the increase in the percentage of women in the studies and the awareness of the HPV vaccine. In numerous studies conducted across different populations in regions like the United States, Europe, and China, it has been found that women’s awareness or knowledge of the HPV vaccine is generally higher than that of men [14,18,179,184,185,187]. The lack of disparity between men and women in the Middle East regarding this issue could be ascribed to the cultural and administrative barriers faced by women in these countries in accessing health-related information. Furthermore, the limited inclusion of male participants in the meta-analysis for HPV vaccine awareness, where only two out of 123 studies focused on men and 84.25% of the total population consisted of women, may have hindered the statistical identification of any potential gender-based differences. Although the HPV vaccine was first approved in 2006, this meta-regression analysis did not demonstrate a statistically significant increase in awareness in studies conducted in recent years. Additionally, when subgroup analysis was conducted on the studies based on the date of the study (post-2017 compared to pre-2018), no significant difference was found between the two groups (40.9% vs. 42.6%, *p* = 0.579). In a meta-analysis conducted among college students in China, the awareness of the HPV vaccine was found to be 29.7% in studies conducted before 2016 and 38.8% in those conducted afterward [179]. A study in the United States found no change in the percentage of participants aware of HPV between 2014 and 2017 [188]. Several factors may have contributed to the stagnation in HPV vaccine awareness in the Middle East over the years. These include the absence of the vaccine in immunization programs across many countries, the comparatively low incidence of cervical cancer in these regions resulting in reduced emphasis on HPV vaccination in healthcare agendas, and the cultural and religious barriers that impede the distribution of vaccine-related information to the public.

In our study, the acceptance of the HPV vaccine was found to be 45.6%. Acceptance of the vaccine for children was 52.8%, with 55.6% for girls and 47.6% for boys. A 2022 meta-analysis in China found that the HPV vaccine intention rate among female university students was 16.7% [17]. Another meta-analysis conducted in China in 2023 found that the pooled prevalence of parents’ acceptance of vaccinating their children was 55.3% [180]. In a meta-analysis conducted in Ethiopia, the acceptance of the HPV vaccine was found to be 42.05%, and another meta-analysis including studies on adolescent girls in the same country reported an acceptance rate of 46.5% [181,189]. In the South East Asia and Pacific region, it has been shown that between 56% and 85% of women have a positive intention to receive the HPV vaccine [15]. In a meta-analysis that included 79 studies from 15 different countries, the acceptance of the HPV vaccine for children was found to be 41.5%, with a range of 0.7%–92.8% [190]. In our meta-analysis, the countries with the highest number of studies, namely Turkey, Saudi Arabia, and the UAE, showed HPV vaccine acceptance percentage of 39%, 51.1%, and 57.7%, respectively. Our analysis has also noted that despite fewer studies in other countries, the range of vaccine acceptance varies between 35.5% and 83.2%.

While HPV vaccine acceptance in the Middle East is found to be similar to that in countries where the vaccine is not included in immunization programs, substantial differences in acceptance rates are evident both within Middle Eastern countries and between Middle Eastern and non-Middle Eastern countries. The accessibility and cost of the vaccine in a country could be significant factors contributing to the differences in vaccine acceptance between countries. In the meta-regression analysis aimed at elucidating factors influencing HPV vaccine acceptance, variables including the proportion of HCWs, the percentage of female participants, participant count, study year, and bias score of the study were utilized. However, this analysis revealed that the model possessed minimal explanatory capacity, and none of the factors were significantly associated with HPV vaccine acceptance. We believe that further investigating the reasons for non-acceptance of the HPV vaccine by individuals in Middle Eastern countries is crucial to understanding this issue.

In most Middle Eastern countries, it is observed that the HPV vaccine is not a part of immunization programs, which correlates with the low level of awareness and acceptance of the vaccine. Furthermore, the incidence of cervical cancer in many of these countries is below the threshold defined by the WHO for eliminating cervical cancer as a significant public health issue, which is 4 per 100,000 [2]. Based on the estimated rates from 2018, it appears that the age-standardized incidence of cervical cancer in all countries in the region is below 7.3 per 100,000 [2]. According to 2023 data, the age-standardized incidence rate of cervical cancer per 100,000 individuals in Turkey is between 2.4 and 5.4, in Qatar 5.1, in Kuwait 3.5, in Israel 5.4, in Bahrain 2.8, in Cyprus 6.1, and in Iran, it ranges from 2.1 to 3.6 [191].

Nevertheless, we believe that enhancing HPV vaccine awareness is crucial for multiple reasons: to pre-empt a potential rise in cervical cancer rates, to ensure that high-risk groups are informed about the vaccine, to foster greater acceptance of the vaccine when integrated into immunization programs, to prevent HPV-related diseases beyond cervical cancer, and particularly to improve women’s access to health information, which in turn can fortify the position of women in Middle Eastern societies. Additionally, conducting studies to understand the reasons behind HPV vaccine refusals in Middle Eastern countries will also help in comprehending the nature of vaccine hesitancy.

This meta-analysis has several limitations. First, the included studies exhibit considerable heterogeneity, possibly due to the varied methodological approaches and population characteristics, which may affect the generalizability of the findings. Second, publication bias, as suggested by Egger’s test, although not substantiated by the trim-and-fill method, cannot be completely ruled out. Third, the majority of studies originated from certain countries within the Middle East, which could result in a regional bias affecting the applicability of the findings to other Middle Eastern countries. Finally, the quality assessment indicates potential methodological flaws within some included studies, which could impact the overall conclusions drawn. The strengths of this article are underscored by its methodological rigor and its pioneering focus on the Middle East, a region with unique health challenges and cultural dynamics. It is the first meta-analysis to consolidate data on HPV vaccine awareness in this geopolitically significant area. Adhering to PRISMA guidelines and PROSPERO registration, the study reflects a commitment to scientific transparency. The inclusion of a substantial number of studies across different Middle Eastern countries provides a broad regional perspective. The systematic approach to quality assessment with the JBI checklist further validates the reliability of the findings. These factors, combined with the novel regional insights, contribute significantly to the understanding of HPV vaccine awareness in the Middle East.

## Supporting information

Gulle et al. supplementary material Gulle et al. supplementary material

## Data Availability

The data that support the findings of this study are included in the supplementary material provided with the manuscript. Further inquiries can be directed to the corresponding author.

## Supplementary material

For supplementary material accompanying this paper visit http://doi.org/10.1017/S0950268824001596. click here to view supplementary material
