# Supplementary material for: Awareness and acceptance of human papillomavirus vaccine in the Middle East: A systematic review, meta-analysis, and meta-regression of 159 studies
Source: Epidemiol Infect. 2024 Dec 10;152:e165. doi: 10.1017/S0950268824001596 (PMC11696605; doi:10.1017/S0950268824001596)
Supplement: Gulle et al. supplementary material [file S0950268824001596sup001.docx]

**Epidemiology and Infection**

**Awareness and Acceptance of Human Papillomavirus Vaccine in the Middle East: A Systematic Review, Meta-Analysis and Meta-regression of 159 Studies**

Bugra Taygun GULLE, Pinar KIRAN, Saadet Goksu CELIK, Zeynep Sedef VAROL, Neslisah SIYVE, Ahmet Naci EMECEN, Hilal DUZEL

**Supplementary Material**

**Detailed Search Strategies**

For Pubmed

("accept"[All Fields] OR "acceptabilities"[All Fields] OR "acceptability"[All Fields] OR "acceptable"[All Fields] OR "acceptably"[All Fields] OR "acceptance"[All Fields] OR "acceptances"[All Fields] OR "acceptation"[All Fields] OR "accepted"[All Fields] OR "accepter"[All Fields] OR "accepters"[All Fields] OR "accepting"[All Fields] OR "accepts"[All Fields] OR ("hesitance"[All Fields] OR "hesitancies"[All Fields] OR "hesitancy"[All Fields] OR "hesitant"[All Fields] OR "hesitate"[All Fields] OR "hesitated"[All Fields] OR "hesitating"[All Fields] OR "hesitation"[All Fields] OR "hesitations"[All Fields]) OR ("awareness"[MeSH Terms] OR "awareness"[All Fields] OR "aware"[All Fields] OR "awarenesses"[All Fields]) OR ("knowledge"[MeSH Terms] OR "knowledge"[All Fields] OR "knowledge s"[All Fields] OR "knowledgeability"[All Fields] OR "knowledgeable"[All Fields] OR "knowledgeably"[All Fields] OR "knowledges"[All Fields]) OR ("accept"[All Fields] OR "acceptabilities"[All Fields] OR "acceptability"[All Fields] OR "acceptable"[All Fields] OR "acceptably"[All Fields] OR "acceptance"[All Fields] OR "acceptances"[All Fields] OR "acceptation"[All Fields] OR "accepted"[All Fields] OR "accepter"[All Fields] OR "accepters"[All Fields] OR "accepting"[All Fields] OR "accepts"[All Fields]) OR "willingness"[All Fields] OR ("attitude"[MeSH Terms] OR "attitude"[All Fields] OR "attitudes"[All Fields] OR "attitude s"[All Fields]) OR ("recognition, psychology"[MeSH Terms] OR ("recognition"[All Fields] AND "psychology"[All Fields]) OR "psychology recognition"[All Fields] OR "recognition"[All Fields] OR "recognitions"[All Fields])) AND ("turkey"[MeSH Terms] OR "turkey"[All Fields] OR "turkey s"[All Fields] OR "turkeys"[MeSH Terms] OR "turkeys"[All Fields] OR ("iran"[MeSH Terms] OR "iran"[All Fields]) OR ("iraq"[MeSH Terms] OR "iraq"[All Fields]) OR ("saudi arabia"[MeSH Terms] OR ("saudi"[All Fields] AND "arabia"[All Fields]) OR "saudi arabia"[All Fields]) OR ("yemen"[MeSH Terms] OR "yemen"[All Fields]) OR ("syria"[MeSH Terms] OR "syria"[All Fields] OR "syria s"[All Fields]) OR ("jordan"[MeSH Terms] OR "jordan"[All Fields]) OR ("united arab emirates"[MeSH Terms] OR ("united"[All Fields] AND "arab"[All Fields] AND "emirates"[All Fields]) OR "united arab emirates"[All Fields]) OR ("israel"[MeSH Terms] OR "israel"[All Fields] OR "israel s"[All Fields]) OR ("cyprus"[MeSH Terms] OR "cyprus"[All Fields]) OR ("middle east"[MeSH Terms] OR ("middle"[All Fields] AND "east"[All Fields]) OR "middle east"[All Fields]) OR "Palestine"[All Fields] OR ("lebanon"[MeSH Terms] OR "lebanon"[All Fields] OR "lebanon s"[All Fields]) OR ("oman"[MeSH Terms] OR "oman"[All Fields]) OR ("kuwait"[MeSH Terms] OR "kuwait"[All Fields] OR "kuwait s"[All Fields]) OR ("qatar"[MeSH Terms] OR "qatar"[All Fields] OR "qatar s"[All Fields]) OR ("bahrain"[MeSH Terms] OR "bahrain"[All Fields]) OR (("middle"[All Fields] OR "middles"[All Fields]) AND "east*"[All Fields]) OR "middle east"[MeSH Terms]) AND ("HPV"[All Fields] OR ("papillomaviridae"[MeSH Terms] OR "papillomaviridae"[All Fields] OR "papillomavirus"[All Fields]) OR ("human papillomavirus viruses"[MeSH Terms] OR ("human"[All Fields] AND "papillomavirus"[All Fields] AND "viruses"[All Fields]) OR "human papillomavirus viruses"[All Fields] OR ("human"[All Fields] AND "papillomavirus"[All Fields]) OR "human papillomavirus"[All Fields]) OR "papillomavirus vaccines"[MeSH Terms] OR "papillomavirus vaccines"[MeSH Terms] OR "papillomavirus vaccines"[MeSH Terms])

For Web of Science

(HPV OR papillomavirus OR human papillomavirus) AND (Turkey OR Iran OR Iraq OR Saudi Arabia OR Yemen OR Syria OR Jordan OR United Arab Emirates OR Israel OR Cyprus OR Middle East OR Palestine OR Lebanon OR Oman OR Kuwait OR Qatar OR Bahrain) AND (acceptance OR hesitancy OR awareness OR knowledge OR acceptability OR willingness OR attitude OR recognition)

**Supplementary table 1.** **The reasons for the exclusion of the eliminated articles**

| **Title** | **Author, year** | **Reason of Exclusion** |
| --- | --- | --- |
| Knowledge and Attitudes toward Human Papillomavirus and Vaccination: A Survey among Nursing Students in Saudi Arabia | Abdelaliem S.M.F et al., 2023 | No quantative results of HPV awareness or acceptance |
| Knowledge and Attitude towards Human Papillomavirus and its Vaccination and Affecting Factors among Nursing and Medical Students: a Questionnaire Study | Akalin A, 2022 | No quantative results of HPV awareness or acceptance |
| Doctors’ Attitudes and Practises Regarding Human Papillomavirus Vaccination: A Qualitative Study | Aksoy H et al., 2023 | No quantative results of HPV awareness or acceptance |
| Knowledge and Attitude of Students Studying at Health Department towards HPV and HPV Vaccination | Aksoy N et al., 2022 | No quantative results of HPV awareness or acceptance |
| Knowledge of Human Papilloma Virus (HPV), HPV-vaccine and Pap Smear among Adult Saudi Women | Al Ghamdi N.H, 2022 | No quantative results of HPV awareness or acceptance |
| Awareness of Risk Factors for Cancer among Omani adults- A Community Based Study | Al-Azri M et al., 2014 | No quantative results of HPV awareness or acceptance |
| Does Seeing What Others Do through Social Media Influence Vaccine Uptake and Help in the Herd Immunity Through Vaccination? A Cross-Sectional Analysis | Al-Hasan A et al., 2021 | No quantative results of HPV awareness or acceptance |
| Awareness of Human Papillomavirus (HPV) Infection, Cervical Cancer (CC), and Vaccine among Females in Sultanate of Oman | Al-Mizra A.M, 2022 | Not published article (poster presentation) |
| Knowledge of Saudi Female University Students Regarding Cervical Cancer and Acceptance of the Human Papilloma Virus Vaccine | Al-Shaikh GK et al., 2014 | No quantative results of HPV awareness or acceptance |
| Effectiveness of Health Education Programme: Level of Knowledge about Prevention of Cervical Cancer among Saudi Female Healthcare Students | Al-Shaikh, G.K et al., 2017 | Not cross-sectional study (intervention study) |
| A Mixed Methods Study of Health Care Professionals' Attitudes towards Vaccination in 15 Countries | Alasmari A et al., 2022 | No quantative results of HPV awareness or acceptance |
| Awareness of Human Papillomavirus among Male and Female University Students in Saudi Arabia | Aldawood E et al., 2023 | No quantative results of HPV awareness or acceptance |
| Determinants of Dentists' Readiness to Assess HPV Risk and Recommend Immunization: A Transtheoretical Model of Change-based Cross-sectional Study of Ontario Dentists | Aldossri M et al., 2021 | not Middle East |
| Awareness of Primary Health Care Physicians about Human Papilloma Virus Infection and its Vaccination: A Cross-sectional Survey from Multiple Clinics in Saudi Arabia | Almughais E.S et al., 2018 | No quantative results of HPV awareness or acceptance |
| The Impact of Education Intervention on Awareness of Cervical Cancer and Barriers to HPV Vaccination among Saudi Pregnant Women | Almutairi S.H et al., 2022 | Not cross-sectional study (intervention study) |
| Acceptability of the COVID-19 Vaccine among Adults in Saudi Arabia: A Cross-Sectional Study of the General Population in the Southern Region of Saudi Arabia | Alqahtani Y.S et al., 2022 | not related HPV |
| Knowledge towards HPV Infection and HPV Vaccines among Syrian Mothers | Alsaad M.A et al., 2012 | Not cross-sectional study (intervention study) |
| Knowledge about Cervical Cancer and Awareness about Human Papillomavirus Vaccination among Medical Students in Jordan | Alsous M.M et al., 2021 | No quantative results of HPV awareness or acceptance |
| Human Papillomavirus and its Vaccination: Knowledge and Attitudes among Female University Students in Saudi Arabia | Altamimi T., 2020 | No quantative results of HPV awareness or acceptance |
| Identification of the Knowledge Level of Students Receiving Health Education about the Human Papilloma Virus, Screening Tests, and Human Papilloma Virus Vaccination | Aslan G et al., 2021 | No quantative results of HPV awareness or acceptance |
| Factors Predicting Mothers' Intention toward Human Papilloma Virus Vaccination of Adolescents: A Cross-sectional Study among Iranian Families | Azh N et al., 2021 | No quantative results of HPV awareness or acceptance |
| The Effect of Educational Intervention Based on Health Belief Model on Beliefs Towards Human Papillomavirus Vaccination in a Sample of Iranian Female Nursing Students | Bayrami R et al., 2021 | Not cross-sectional study (intervention study) |
| Does Group Education Affect Mothers' Knowledge and Attitudes Towards the HPV Vaccine? | Bilgin N.C et al., 2022 | Not cross-sectional study (intervention study) |
| Attitudes of Physicians Concerning Vaccines not Included in the National Immunization Schedule | Cataklı T et al., 2018 | No quantative results of HPV awareness or acceptance |
| Awareness of Human Papillomavirus and Human Papillomavirus Vaccines in Cypriot Population | Cerit Z et al., 2018 | full text unavailable |
| What do Lebanese Women Know about Cervical Cancer and Human Papillomavirus? A report on Awareness Levels in Urban Communities | Choucair, J.E, et al., 2018 | Not published article (poster presentation) |
| HPV and HPV Vaccination: Knowledge and Consciousness of Young Women | Cosar E et al., 2014 | No quantative results of HPV awareness or acceptance |
| The Effects of Two Different Teaching Techniques on the Knowledge Level of Nursing Students about HPV | Dag H et al., 2015 | Not cross-sectional study (intervention study) |
| Human Papillomavirus Knowledge and Vaccine Acceptability in Jazan Province, Saudi Arabia | Darraj A.I et al., 2022 | No quantative results of HPV awareness or acceptance |
| Maternal Factors Affecting HPV Vaccine Acceptance for their Adolescent Children Aged between 10 and 15 | Dursun P. Et al., 2009 | Not original data |
| Enhancing the Knowledge of Cervical Cancer Screening among Female Nursing Students: An Interventional Educational Program | Eittah, H.F.A et al., 2020 | Not cross-sectional study (intervention study) |
| Effects of Information Sources in HPV Vaccine Acceptance: Prospective Randomized Trial | Ekmez F et al., 2022 | Not cross-sectional study (intervention study) |
| Recommending Immunizations to Adolescents in Turkey: a Study of the Knowledge, Attitude, and Practices of Physicians | Elitok G.K et al., 2020 | No quantative results of HPV awareness or acceptance |
| We Get Vaccinated, But We Do Really Know Why?: Evaluation on Knowledge on HPV Infection and Vaccination in Medical School Students with HPV Vaccine | Erdem H.A et al., 2020 | HPV vaccinated population |
| The Effect of University Students' Levels of Knowledge about HPV Infection and the HPV Vaccine on Their Health Beliefs: Health Sciences Students | Ergun S, 2023 | No quantative results of HPV awareness or acceptance |
| Prevention of Gynecological Cancers: The Affecting Factors and Knowledge Levels of Turkish Women | Evcili, Funda et al., 2019 | No quantative results of HPV awareness or acceptance |
| Awareness of HPV and Cervical Cancer Prevention among University Health Sciences Students in Cyprus | Farazi P.A et al., 2019 | No quantative results of HPV awareness or acceptance |
| Barriers to Human Papillomavirus Vaccine Acceptability in Israel | Fisher W.A et al., 2013 | Not research article (review) |
| School-Located Vaccination Clinics for Adolescents: Correlates of Acceptance among Parents | Gargano L.M et al., 2014 | not Middle East |
| Voluntary or Mandatory? The Valence Framing Effect of Attitudes Regarding HPV Vaccination | Gesser-Edelsburg A et al., 2015 | No quantative results of HPV awareness or acceptance |
| Parental Knowledge and Attitudes about Human Papilloma Virus in Iran | Ghojazadeh M, 2012 | No quantative results of HPV awareness or acceptance |
| Post-conization Follow-up of Patients with CIN 2/3 with Different Amount of Distance to Negative Cone Biopsy Margin: a Retrospective Cohort Study | Giray B et al., | Not cross-sectional study (cohort study) |
| A survey on Prevalence, Risk Factors and Knowledge for Oral Human Papilloma Virus Infections among University Students | Guran, Mumtaz et al., 2021 | No quantative results of HPV awareness or acceptance |
| A case-control Study to Evaluate Awareness Level of Human Papillomavirus among Women Healthcare Professionals in Tertiary Health Care Facility | Guven S.E et al., 2021 | Not cross-sectional study (case-control study) |
| Health Belief Model Scale for Human Papilloma Virus and its Vaccination: Adaptation and Psychometric Testing | Guvenc G et al., 2016 | Not cross-sectional study (methodological study) |
| Awareness about the human papillomavirus (HPV) and HPV vaccine among medical students in Lebanon | Haddad S.F et al., 2021 | No quantative results of HPV awareness or acceptance |
| The Impact of Teachings on Sexuality in Islam on HPV Vaccine Acceptability in the Middle East and North Africa Region | Hamdi S, 2018 | Not research article (review) |
| Saudi Women Health Beliefs and Associated Factors Regarding Cervical Cancer Prevention at Najran city: A Theory-based Study | Ibrahim, Heba A et al., 2022 | No quantative results of HPV awareness or acceptance |
| Determinants of Human Papillomavirus Vaccine Recommendation among Middle Eastern and Lebanese Healthcare Providers | Jaafar I et al. 2022 | No quantative results of HPV awareness or acceptance |
| Barriers, Attitudes and Clinical Approach of Lebanese Physicians Towards HPV Vaccination | Jaude J.A et al., 2019 | No quantative results of HPV awareness or acceptance |
| Knowledge, Attitudes, and Practices among Saudi Women Regarding Cervical Cancer, Human Papillomavirus (HPV) and Corresponding Vaccine | Jradi H et. al, 2019 | No quantative results of HPV awareness or acceptance |
| Prospects and Challenges in the Introduction of Human Papillomavirus Vaccines in the Extended Middle East and North Africa Region | Jumaan A.O et al., 2013 | Not research article (review) |
| The Study of the Knowledge Levels and Awareness of the Academicians Who Are Not in the Field of Health in Relation to Cervical Cancer | Karabulutlu O et al., 2016 | No quantative results of HPV awareness or acceptance |
| Assessment of Knowledge Level and Awareness about Human Papillomavirus among Dental Students | Keser G et al., 2021 | No quantative results of HPV awareness or acceptance |
| Human Papillomavirus Vaccine Knowledge and Conspiracy Beliefs among Secondary School Students in Lebanon | Khalil J et al., 2023 | No quantative results of HPV awareness or acceptance |
| Knowledge of Human Papillomavirus (HPV), Attitudes, and Practices towards Anti-HPV Vaccination among Israeli Nurses | Khamisy-Farah R et al., 2023 | No quantative results of HPV awareness or acceptance |
| Knowledge of Human Papillomavirus (HPV), Attitudes and Practices Towards Anti-HPV Vaccination Among Israeli Pediatricians, Gynecologists, and Internal Medicine Doctors: Development and Validation of an Ad Hoc Questionnaire | Khamisy-Farah R, et al., 2019 | Not cross-sectional study (methodological study) |
| The Impact of Education About Cervical Cancer and Human Papillomavirus on Women's Healthy Lifestyle Behaviors and Beliefs Using the PRECEDE Educational Model | Koc Z et al, 2019 | Not cross-sectional study (intervention study) |
| Vaccination Coverage Against Human Papillomavirus in Female Students in Cyprus | Konstantinou C et al., 2022 | No quantative results of HPV awareness or acceptance |
| A survey of Jordanian Obstetricians and Gynecologists' Knowledge and Attitudes toward Human Papillomavirus Infection and Vaccination | Latafieh I et al., 2014 | No quantative results of HPV awareness or acceptance |
| Perspectives of Medical Experts' on Health Culture for Human Papillomavirus (HPV) Vaccination Compared to Auxiliary Health Workers | Masjedi et al., 2022 | No quantative results of HPV awareness or acceptance |
| Patients' Knowledge and Attitudes Regarding Human Papillomavirus Disease and the Effectiveness of Education in Patients referred to the Dermatology Clinic of Baqiyatallah Hospital in 2020 | Molaei H et al., 2022 | Not cross-sectional study (intervention study) |
| The Evaluation of the Knowledge Levels and Attitudes of Medicaal Students Who Have Accomplished Obstetric and Gynaecological Diseases Internship in a Medical School about Human Papilloma Virus Vaccine | Onsuz M.F et al., 2011 | No quantative results of HPV awareness or acceptance |
| Awareness and Knowledge about Human Papillomavirus Infection and Vaccination among Women in UAE | Ortashi O et al., 2013 | Not original data |
| Need Assessment about Cervical Cancer Prevention Trainings among Woman Health Care Workers Working in a Training and Research Hospital in Turkey | Ozturk O et al., 2018 | No quantative results of HPV awareness or acceptance |
| Evaluation of Opinions of Pediatrists in a Tertiary Healthcare Hospiatal About Meningococcal, Rotavirus, Human Papilloma Virus Vaccines and İncidence of Encountering Vaccine Refusal and Reasons of Vaccine Refusal | Parlakay A.O et al., 2020 | No quantative results of HPV awareness or acceptance |
| Human Papillomavirus (HPV): Unawareness of the Causal Role of HPV Infection in Cervical Cancer, HPV Vaccine Availability, and HPV Vaccine Uptake among Female Schoolteachers in a Middle Eastern Country | Rezqalla J et al., 2021 | No quantative results of HPV awareness or acceptance |
| Overview of Cervical Cancer Screening Practices in the Extended Middle East and North Africa Countries | Sancho-Garnier H et al., 2013 | Not research article (review) |
| Cost-Benefit Analysis of Human Papillomavirus Vaccine in Iran | Sargazi N et al., 2022 | No quantative results of HPV awareness or acceptance |
| Prevalence of HPV Genotypes in South Europe: Comparisons between an Italian and a Turkish Unvaccinated Population | Schettino et al., 2019 | Not cross-sectional study (cohort study) |
| Awareness, Attitudes, Practices and Continuation of HPV Vaccines in Lebanon | Seoud M et al., 2018 | Not published article (poster presentation) |
| Outcomes of Human Papilloma Virus Vaccination in a Private Women Health Clinic in Lebanon | Seoud, Muhieddine et al., 2022 | No quantative results of HPV awareness or acceptance |
| Case of Paradoxical Cultural Sensitivity: Mixed Method Study of Web-Based Health Informational Materials About the Human Papillomavirus Vaccine in Israel | Shahbari N.A.E et al., 2019 | No quantative results of HPV awareness or acceptance |
| The Effectiveness of a Model-based Health Education Program on Protective Behavior against Human Papillomavirus in Female Drug abusers: A randomized Controlled Trial | Shokoohi M et al, 2019 | Not cross-sectional study (intervention study) |
| Knowledge about the Risk Factors for Cervical Cancer and Human Papillomavirus Vaccine among Nursing Students | Tas F et al., 2008 | Not published article (poster presentation) |
| Utah Pharmacists' Knowledge, Attitudes, and Barriers Regarding Human Papillomavirus Vaccine Recommendation | Tolentino V et al., 2018 | not Middle East |
| Effectiveness of an Educational Intervention to Increase Human Papillomavirus Knowledge and Attitude in Staff and Nursing Students | Torabizadeh C et al., 2020 | Not cross-sectional study (intervention study) |
| Awareness on HPV and HPV Vaccine and Acceptance of Vaccination against HPV among High School Students and Their Parents: A Private High School Based Study | Tulpar B et al., 2017 | Not published article (poster presentation) |
| Individualism, Acceptance and Differentiation as Attitude Traits in the Public's Response to Vaccination | Velan B et al., 2012 | No quantative results of HPV awareness or acceptance |
| On the Implications of Desexualizing Vaccines against Sexually Transmitted Diseases: Health Policy Challenges in a Multicultural Society | Velan, Baruch et al., 2017 | No quantative results of HPV awareness or acceptance |
| Correlations of HPV Vaccine Uptake among Eight-grade Students in Israel: the Importance of Ethnicity and Level of Religious Observance | Wortsman J et al., 2023 | No quantative results of HPV awareness or acceptance |
| Assessing the Role of Education on Turkish University Students' Knowledge about HPV and Related Diseases | Yanikkerem E et al., 2010 | Not cross-sectional study (intervention study) |
| Knowledge, attitudes, practices and barriers towards HPV vaccination among nurses in Turkey: a longitudinal study | Yanikkerem E et al., 2014 | Not cross-sectional study (intervention study) |
| Designing and Psychometric Assessment of the Scale of Factors Influencing HPV Vaccine Uptake Behaviors in Young Adults | Yarmohammadi S et al., 2022 | Not cross-sectional study (methodological study) |
| Knowledge and Attitudes of the Pediatricians in Turkey Regarding Human Papillomavirus (HPV) Vaccine | Yildirim M et al., 2009 | No quantative results of HPV awareness or acceptance |
| Determination of Knowledge Levels, Attitude and Behaviors of Female University Students Concerning Cervical Cancer, Human Papiloma Virus and its Vaccine | Yoruk S et al., 2016 | No quantative results of HPV awareness or acceptance |
| Relationship Between Awareness of Cervical Cancer and HPV Infection and Attitudes towards HPV Vaccine among Women Aged 15-49 Years: a Cross-sectional Study | Yurtcu E et al., 2022 | No quantative results of HPV awareness or acceptance |
| Knowledge and Practice of Pap Smear and Vaccination Regarding Human Papillomavirus Among Female Medical Students in Karachi, Pakistan | Zaidi, T.H et al., 2021 | not Middle East |
| Opportunities to Accelerate Immunization Progress in Middle-income Countries | Zhu J et al., 2023 | not Middle East |
| The Effect of Education Based on the Theory of Planned Behavior on the Intention of Vaccination against Human Papillomavirus in Female Students: A Controlled Educational Trial | Zomordi G et al., 2022 | Not cross-sectional study (intervention study) |
| Knowledge of Human Papillomavirus and Acceptability to Vaccinate in Adolescents and Young Adults of the Moroccan Population | Zouheir, Yassine et al., 2016 | not Middle East |

**Supplementary table 2. Characteristics of included studies**

| **First author, year** | **Country*** | **Number**  **of participants** | **Population** | **Study enrollment time (year)** | **Female (%)** | **Age**(mean±std)** | **Data collection method** | **Awareness (%)** | **Acceptance (%)** | **Acceptance for girl (or for child) (%)***** | **Acceptance for boy (%)** |
| --- | --- | --- | --- | --- | --- | --- | --- | --- | --- | --- | --- |
| Abou El-Ola M.J et al., 2018 | Lebanon | 1193 | mothers of schoolgirls | 2016-2017 | 100 | 42.6 ±5.8 | self administered questionaire | 34.2 |  |  |  |
| Abuduzike G et al., 2022 | Cyprus | 227 | parents | 2019 | 70 | 34.6 | face to face | 45.4 |  | 55.7 (child) |  |
| Acar O.N et al., 2019 | Turkey | 270 | physicians and nurses |  | 63.3 | 42.2±6.5 | self administered questionaire |  |  | 58.5 |  |
| Acoglu E.A et al., 2019 | Turkey | 203 | parents with children aged 9-18 years | 2018-2019 | 79.8 | 39.1±6.1 | face to face | 29.1 |  | 62.1 |  |
| Adiguzel F.I et al., 2016 | Turkey | 426 | women applied gynecology clinic | 2015 | 100 | 32.3±10.1 | self administered questionaire | 33.1 | 42.7 | 43.7 (child) |  |
| Aga S.S et al., 2022 | Saudi Arabia | 580 | health professions students | 2020 | 77.9 | 20.4±1.7 | online | 34.1 |  |  |  |
| Akca G et al, 2022 | Turkey | 330 | mothers who applied pediatric outpatient clinic | 2022 | 100 | 36.1 (19-65) | face to face | 33.9 | 67.3 |  |  |
| Akkour K et al., 2021 | Saudi Arabia | 564 | women | 2020 | 100 | 25-34 (29.8) | online | 17.7 | 54.1 |  |  |
| Akyuz A et al., 2011 | Turkey | 229 | women applied gynaecology clinic | 2007-2008 | 100 | 37.8±8.3 | face to face | 72.1 |  |  |  |
| Al Darwish A.A et al., 2014 | Saudi Arabia | 188 | medical students | 2012-2013 | 40.9 |  | self administered questionaire | 33.9 |  |  |  |
| Al Raisi M et al., 2021 | Oman | 805 | women | 2019-2020 | 100 | 31-40 (40.6) | face to face | 10.1 |  | 47 |  |
| Al-Nuaimi N.S et al, 2011 | Saudi Arabia | 334 | female highschool students |  | 100 | 17 (15-20) | self administered questionaire |  | 76.7 |  |  |
| Al-Saadi A.N et al., 2021 | Oman | 791 | women attending primary healthcare centres | 2018-2019 | 100 | 31-40 (48) | face to face | 21 | 73.8 |  |  |
| Alawi S.A et al., 2023 | Oman | 1403 | adults | 2021 | 69.1 | 26-35 (41.0) | online |  | 58 |  |  |
| Aldhafar A.S et al., 2016 | Saudi Arabia | 290 | female teachers | 2015 | 100 | 36.4 |  | 10 |  |  |  |
| Aldohaian A.I et al., 2019 | Saudi Arabia | 450 | adult women | 2018 | 100 | 32.9 ± 8.3 | face to face | 9.9 |  |  |  |
| AlHarfi M.M.A al., 2019 | Saudi Arabia | 242 | married women | 2019 | 100 | 27.5±12.5 | face to face | 21.1 | 61.7 |  |  |
| Alhusayn K.O et al., 2022 | Saudi Arabia | 296 | parents | 2020 | 80.7 | 31-40 (46.9) | face to face | 29.4 | 44 |  |  |
| Ali M.D et al., 2022 | Saudi Arabia | 125 | undergraduate pharmacy students | 2020 | 87.2 | 18-25 (84) | online | 65 |  |  |  |
| Alkalash S.H et al., 2022 | Saudi Arabia | 343 | parents | 2022 | 66.2 | 36-45 (39.1) | online | 32.9 |  | 58.6 (for child) |  |
| Alkan S et al.,2021 | Turkey | 224 | college students | 2021 | 84,8 | 20,5±1.2 | online | 47.3 |  |  |  |
| Alkhaldi R.O et al., 2023 | Saudi Arabia | 624 | women between 18 and 50 years | 2020-2021 | 100 | 20-30 (48.4) | online |  | 75.8 |  |  |
| Almaghlouth A.K et al., 2022 | Saudi Arabia | 644 | Saudi citizens | 2022 | 64 | 28.8±11.4 | online | 35.2 | 47.7 |  |  |
| Almazroua S et al., 2020 | Saudi Arabia | 173 | pediatricians and family medicine physicians | 2017-2018 | 50.9 | <30 (44.5) | online | 84.4 |  | 82.1 |  |
| Almehmadi M.M et al., 2019 | Saudi Arabia | 1022 | general population | 2018-2019 | 65.5 | 15-22 (56) | online | 17 |  |  |  |
| Alnafisah R.A et al., 2019 | Saudi Arabia | 2220 | women | 2018-2019 | 100 | 31-45 (42.9) | self administered questionaire |  |  | 70.8 |  |
| Alqarawi S.A et al., 2023 | Saudi Arabia | 387 | women | 2023 | 100 | 18-25 (52.2) | self administered questionaire |  | 54 |  |  |
| Alshammiri S.M, 2022 | Saudi Arabia | 386 | female married teacher | 2021 | 100 | 41.4 ± 5.3 | self administered questionaire |  |  | 38 |  |
| Alsanafi M et al., 2023 | Kuwait | 610 | university students | 2022 | 100 | <22 (45) | online | 43.5 | 38.6 |  |  |
| Alshammari F et al., 2022 | Saudi Arabia | 386 | university students | 2020 | 37 | 24±7.1 | self administered questionaire | 30.8 |  |  |  |
| Alshehri A.M et al., 2021 | Saudi Arabia | 550 | adults | 2021 | 30.4 | 47.5 (11.5) |  |  | 30 |  |  |
| Alsous M.M et al., 2021 | Jordan, Iraq, UAE, Qatar | 2804 | women | 2020 | 100 | 18-25 (35.9 ) | online | 26.1 | 43.2 |  |  |
| Anfinan N.M, 2019 | Saudi Arabia | 2000 | physicians | 2017-2018 | 52.8 | 31–40 (30.4) | self administered questionaire |  | 41.2 | 77.6 (for child) |  |
| Ates S.A et al., 2020 | Turkey | 151 | healthcare workers | 2020 | 68.9 | 40-49 (43.7) | face to face | 29.8 | 11.9 | 17.9 (for child) |  |
| Aynacı G et al., 2019 | Turkey | 396 | undergraduate university students | 2018 | 62.1 | 18-29 (100) | self administered questionaire | 54.8 |  |  |  |
| Azer S.A et al., 2022 | Saudi Arabia | 172 | college students | 2022 | 49.4 |  | online | 58.7 | 43.6 |  |  |
| Bal-Yılmaz H et al., 2018 | Turkey | 624 | nursing student | 2015 | 81.4 | 20.7 ±2.0 | self administered questionaire |  | 14.4 |  |  |
| Bantun F et al., 2022 | Saudi Arabia | 214 | female patients 18-60 years old | 2022 | 100 | 30> (67.2) | online | 32.7 |  |  |  |
| Barhamain A.S et al., 2022 | Saudi Arabia | 609 | adult women | 2021-2022 | 100 | 33 (IQR:13) | online | 36 |  | 86.5 |  |
| Basar F et al., 2019 | Turkey | 216 | nursing students |  | 79.6 | 20.66±2.6 | self administered questionaire | 42.6 |  |  |  |
| Baser E et al., 2019 | Turkey | 553 | patients referred gynecology clinic |  | 100 | 30> (51) | face to face | 9.8 | 56.6 | 58.6 | 59.3 |
| Borlu A et al., 2016 | Turkey | 718 | college students | 2011-2012 | 66.1 |  | self administered questionaire | 36.1 | 8.8 |  |  |
| Bulbul S et al., 2013 | Turkey | 1405 | mothers | 2007 | 100 | 31.2±7.0 | face to face | 46.3 | 25.5 |  |  |
| Celik P et al., 2021 | Turkey | 1000 | parents with children aged 9-18 years | 2017-2018 | 76.2 | 37.7±6.4 | face to face | 22 |  | 8.1 (for child) |  |
| Cenk H et al., 2022 | Turkey | 806 | medical students | 2021 | 59 | 21.6±2.0 | online | 74 | 79.5 | 87.4 | 81.2 |
| Cetin O et al., 2014 | Turkey | 501 | adolescent girls | 2012-2013 | 100 | 15.9±1.4 | self administered questionaire | 11.7 | 6.3 |  |  |
| Charalambous I et al., 2019 | Cyprus | 283 | female college students | 2017 | 100 | 19.7±1.5 | self administered questionaire |  | 30.7 |  |  |
| Christodoulou A et al., 2019 | Cyprus | 200 | female healthcare workers | 2015 | 100 | 20-29 (35) | self administered questionaire | 81.7 | 83.5 | 82.9 | 79.1 |
| Cimke V.S et al., 2018 | Turkey | 753 | adult women |  | 100 | 34.7 ± 7.5 | face to face | 61.3 |  |  |  |
| Cinar I.O et al., 2019 | Turkey | 1563 | college student | 2018 | 42.5 | 22.3±2.1 | self administered questionaire | 7.3 |  |  |  |
| Dany M et al. 2015 | Lebanon | 215 | female students | 2013-2014 | 100 | 22.7 ±4.3 | self administered questionaire | 63.5 |  |  |  |
| Donmez S et al., 2019 | Turkey | 690 | female nursing students | 2016 | 100 | 20.4 ±3.0 | self administered questionaire | 17.4 | 25.4 |  |  |
| Dursun P et al., 2009 | Turkey | 1427 | women | 2007 | 100 | 35.8 ±10.8 | face to face |  | 70 | 64 | 59 |
| Durusoy R et al., 2010 | Turkey | 717 | preparatory class students | 2010 | 62.1 | 19.7±1.5 | self administered questionaire | 25.1 | 10.9 |  |  |
| Elnashar M.A et al., 2022 | Saudi Arabia | 352 | parents | 2021-2022 | 90.1 | 30-50 (50) | online | 26.5 |  | 52.7 (for child) |  |
| Elshami M et al., 2022 | Palestine | 7058 | non-health related women | 2019-2020 | 100 | 32 [24.0- 42.0] | face to face | 0.65 |  |  |  |
| Erbaydar N et al., 2016 | Turkey | 246 | nurses | 2012 | 100 | 34.2±8 | self administered questionaire |  | 33.8 | 36.6 |  |
| Erbiyik H.I et al., 2021 | Turkey | 700 | healthcare workers, studtens and patients | 2019 | 76.6 | 18-29 (79.6) | self administered questionaire | 54.0 | 38.3 |  |  |
| Erdogan M et al., 2021 | Turkey | 250 | women applied cancer screening center | 2015 | 100 | 47.7±7.5 | face to face | 20.8 |  | 94.4 |  |
| Farsi N.J et al., 2020 | Saudi Arabia | 484 | dental students | 2018-2019 | 65 | 11-26 (100) | self administered questionaire | 57 | 45 |  |  |
| Farsi N.J et al., 2021 | Saudi Arabia | 517 | male medical student | 2017-2018 | 0 | 21 ±1.4 | self administered questionaire | 58.4 | 48.9 |  |  |
| Genc R.E et al., 2013 | Turkey | 268 | midwifery students | 2011 | 100 | 20.8±1.6 | self administered questionaire | 40.4 |  |  |  |
| Gokmen-Karasu A.F et al., 2019 | Turkey | 499 | nurses | 2016 | 70.7 | 25.9 ±6.1 |  |  | 44.3 | 52.8 (for child) |  |
| Gol I et al., 2016 | Turkey | 110 | female nurses | 2005-2016 | 100 | 25-29 (29.1) | face to face | 54.5 |  |  |  |
| Gorkem U et al., 2015 | Turkey | 192 | women healthcare workers | 2015 | 100 | 36.1±7,9 | face to face | 85.4 | 58.3 | 74,5 |  |
| Gunes O et al., 2023 | Turkey | 564 | parents and healtcare workers parents |  | 66.9 | 39.7±10.7 | face to face | 66.1 |  | 42.2 (for child) |  |
| Gursoy M.Y et al., 2023 | Turkey | 1723 | male student | 2022 | 0 | 21.7±2.2 | online |  | 10.4 |  |  |
| Guder S et al.,2022 | Turkey | 209 | healthcare workers | 2022 | 70,8 | 36.0 ±9.3 | face to face | 95.7 | 57.4 | 85.6 (for child) |  |
| Guducu N et al., 2012 | Turkey | 603 | medicine students of the first three years, nursing students and nurses | 2010-2011 | 79.8 | 25.4±6.4 | self administered questionaire |  |  | 52.1 |  |
| Guvenc G et al., 2012 | Turkey | 314 | nursing students | 2010 | 100 | 20.26±1.14 | self administered questionaire | 55.4 | 28.0 |  |  |
| Hashemipour M.A et al., 2019 | Iran | 290 | medical and dental students | 2017 | 55.5 | 29-37 (medians of two different groups) | self administered questionaire | 60.1 | 62.1 |  |  |
| Hendaus M.A et al., 2021 | Qatar | 334 | parents | 2021 | 75 | 30-39 (54.5) | self administered questionaire |  |  | 80.5 (for child) |  |
| Husain Y et al., 2019 | Bahrain | 408 | men and women aged 18–65 | 2018 | 65.7 | 34.5±9.7 | face to face |  | 76 |  |  |
| Hussain A.N et al., 2016 | Saudi Arabia | 325 | women | 2012-2014 | 100 | 17.1±5.3 | face to face | 32.3 | 64 |  |  |
| Ilter E et al., 2010 | Turkey | 525 | women attending a gynecology clinic | 2009 | 100 | 32.3 ± 9.6 | face to face | 77 | 56 | 89 |  |
| Jassim G et al., 2018 | Bahrain | 300 | females attending the primary health care centres | 2015-2016 | 100 | 37.2±11.9 | face to face | 3.7 | 81.8 | 90.9 (for child) |  |
| Karaoglu S.A et al., 2022 | Turkey | 182 | adults | 2018 | 51.1 | 32.9±12.8 | face to face | 29.6 |  |  |  |
| Kayi I et al., 2020 | Turkey | 1115 | female college students | 2015 | 100 | 20.8±2.0 | face to face | 75.2 | 35.8 |  |  |
| Kazanci F et.al., 2022 | Turkey | 500 | high school and university students | 2018-2019 | 50.8 | 19.8 (14-26) | face to face | 58.8 |  |  |  |
| Keser G et al., 2020 | Turkey | 226 | dental students |  | 66.8 | 23.2±1.3 |  |  | 41.6 |  |  |
| Keten H.S, et al., 2021 | Turkey | 804 | teachers | 2015 | 44.3 | 38.3 ± 8.4 | face to face | 25.7 | 18.7 |  |  |
| Khoury J.E et al., 2023 | Lebanon | 454 | female students aged 17-30 | 2021 | 100 | 22.7±3.33 | online | 66.7 | 52.0 |  |  |
| Kilicaslan O et al., 2021 | Turkey | 1023 | mothers of pediatric patients | 2018 | 100 | 40±6 | self administered questionaire | 21.6 |  | 30.9 | 36.2 |
| Kiskac N et al., 2021 | Turkey | 1425 | adult |  | 59.6 | 37.4±10.8 | online | 50.4 |  |  |  |
| Kizmaz M et al., 2022 | Turkey | 409 | women aged 15-49 | 2019-2020 | 100 | 30.0±9.1 | self administered questionaire | 49.4 | 31.9 |  |  |
| Kilic A et al., 2012 | Turkey | 405 | nursing students and parents | 2011-2012 | 66.6 | 37.1±3.7 | self administered questionaire |  | 37.7^1^ | 45.2^2^ |  |
| Koc Z et.al., 2015 | Turkey | 464 | nurses | 2013 | 100 | 31.8±6.0 | self administered questionaire | 63.8 | 17.5 | 30.2 (for child) |  |
| Koc Z, 2014 | Turkey | 800 | female college students | 2012 | 100 | 20.4±1.6 | face to face | 8.7 | 32.6 |  |  |
| Kose D et al., 2014 | Turkey | 799 | mothers | 2014 | 100 | 32.0±6.5 | face to face | 16.5 |  |  |  |
| Kurtipek G.S et al., 2016 | Turkey | 543 | women who applied derrmatology outpatient clinic | 2016 | 100 | 31.9±10.9 | self administered questionaire | 32 |  | 32 |  |
| Kurtoğlu E. et. Al. 2013 | Turkey | 53 | family physician |  | 43.4 | 37.7±5.9 | face to face | 77.4 | 50.9 | 32.1 | 26.4 |
| Lataifeh I et al., 2016 | Jordan | 339 | female university students | 2014 | 100 |  | self administered questionaire | 45.7 |  |  |  |
| Levent C et al., 2021 | Turkey | 299 | mothers or fathers | 2019 | 91.3 | 38.9±4.9 | face to face | 15.7 |  | 30.8 (for child) |  |
| Lingam A.S et al., 2022 | Saudi Arabia, UAE, Egypt | 393 | dental students | 2021 | 73.1 |  | online | 34.9 |  |  |  |
| Mahomed, M.F et al., 2020 | Saudi Arabia | 319 | not single women | 2017 | 100 | 31-40 (33.5) | online | 11.6 |  |  |  |
| Mete B et al., 2023 | Turkey | 401 | women | 2022 | 100 | 30.9±8.9 | face to face | 74.1 | 81.8 | 72.8 (child) |  |
| Mojahed S. et al., 2013 | Iran | 357 | nursing staff | 2013 | 100 |  | face to face |  | 41.2 |  |  |
| Moosa K et al., 2014 | Bahrain | 571 | women | 2010-2011 | 100 | 35.6±11.2 | face to face | 31.3 | 91.3 |  |  |
| Naki M.M et al., 2016 | Turkey | 311 | healthcare workers |  | 67.2 |  | face to face |  | 55 | 74.6 | 49.8 |
| Natan M.B et al., 2011 | Israel | 103 | mothers | 2011 | 100 | 41.3 ±9.4 | self administered questionaire |  | 65 |  |  |
| Onan A et al., 2009 | Turkey | 1808 | female | 2007 | 100 | 37.6±13.0 | face to face | 24.3 | 17 | 23.6 |  |
| Onder O. et al., 2015 | Turkey | 294 | women |  | 100 | 30.5±8.9 | self administered questionaire | 24.5 |  |  |  |
| Ortashi O et al., 2012 | UAE | 125 | nurses | 2012 | 100 | <30 (40) | face to face | 96.8 | 74.4 |  |  |
| Ortashi O et al., 2013 | UAE | 356 | male university students | 2012 | 0 | 21±1.5 | self administered questionaire | 31 | 46 |  |  |
| Ortashi O et al., 2014 | UAE | 640 | women | 2012 | 100 | 32.4±8.3 | face to face | 37 | 79.5 |  |  |
| Oz M et al., 2016 | Turkey | 1160 | college students aged 18–30 |  | 58.3 | 22.1[[18-30]] | online | 26.6 | 45.1 |  |  |
| Ozcam H et al., 2014 | Turkey | 200 | women healthcare workers | 2013 | 100 | 36.5±9.5 | self administered questionaire | 72.0 |  |  |  |
| Ozdede M et al., 2020 | Turkey | 209 | dentists and dental students | 2020 | 75.1 | 25.7^2^ | self administered questionaire | 95.2 |  |  |  |
| Ozsurekci Y et al., 2013 | Turkey | 226 | turkish pediatricians | 2011 | 52 | 34.4±8.7 | self administered questionaire |  |  | 75 |  |
| Ozyer S et al., 2013 | Turkey | 408 | female aged 9-24 |  | 100 | 18.5±2.7 | self administered questionaire | 21 | 11.2 |  |  |
| Parlak E et al., 2021 | Turkey | 237 | non-health related women | 2019 | 100 | 33.9±10.5 | face to face |  | 28.3 | 34.2 | 30.4 |
| Purut Y.E et al., 2020 | Turkey | 197 | HPV (+) patients | 2018-2019 | 100 | 38.60±8.78 | face to face | 56.9 | 13.7 | 13.7 |  |
| Rathfisch G et al., 2015 | Turkey | 605 | undergraduate students |  | 73.7 | 20.4 | self administered questionaire | 44.5 | 39.5 | 39.8 |  |
| Revanli R.A et al., 2016 | Turkey | 263 | family physician | 2014 | 16.2 | 43.3 | face to face | 89.3 |  |  |  |
| Sabr Y et al., 2021 | Saudi Arabia | 382 | female patients | 2018-2019 | 100 | 26-45 (70.6) | self administered questionaire | 12.0 | 29.3 | 41.1 |  |
| Sahin H.G et al., 2017 | Turkey | 1000 | outpatient women | 2013 | 100 | 42.1 | face to face | 1.3 | 23.2 |  |  |
| Sahin H.O et al., 2020 | Turkey | 199 | medical students | 2019 | 58.8 | 22.3±2.1 | self administered questionaire |  | 43.6 | 71.9 (for child) |  |
| Sahin R.A et al., 2022 | Turkey | 561 | college students | 2019-2020 | 62.9 | 21.4±2.0 | face to face |  | 42.6 |  |  |
| Sait K.H, 2009 | Saudi Arabia | 500 | not single women | 2008 | 100 | 42 (median) | self administered questionaire | 9.8 |  |  |  |
| Sallam M et al., 2021 | Jordan | 836 | female university students with health related | 2020-2021 | 100 | 20–22 (53.8) | self administered questionaire | 48.7 | 75 |  |  |
| Sallam M et al., 2022 | Iraq | 1198 | medical students | 2019 | 58.2 | 20.7±1.7 | face to face | 65.5 |  |  |  |
| Saqer A et al., 2017 | Saudi Arabia | 400 | parents | 2015 |  | 40.6±10.4 | self administered questionaire | 36.5 |  | 83.2 |  |
| Sargazi N et al., 2021 | Iran | 306 | mothers | 2019 | 100 | 31-40 (67.3) | face to face |  |  | 43 |  |
| Senol V et al., 2011 | Turkey | 1000 | married women | 2009 | 100 | 25-44 (55.5) | face to face | 11 |  |  |  |
| Seven M et al., 2015 | Turkey | 736 | primary school parents | 2013-2014 | 50 | 36.5 | self administered questionaire | 22.9 |  | 15 | 22 |
| Shdefat S.A et al., 2022 | United Arab Emirates | 390 | men |  | 0 |  | self administered questionaire |  | 37.7 |  |  |
| Shibli R et al., 2019 | Israel | 313 | mothers of 8th grade students | 2016-2017 | 100 | 42.9 ±4.8 | telephone | 84 | 62.9 |  |  |
| Sundaram M.K et al., 2021 | United Arab Emirates | 269 | female expatriate university students |  | 100 | 18-26 (100) |  | 38.6 |  |  |  |
| Tas B et al., 2016 | Turkey | 273 | outpatients | 2016 | 33 | 26-39 (52.6) | face to face |  | 54.2 |  |  |
| Tas F et al., 2010 | Turkey | 400 | nursing student |  | 100 | 21.5±1.7 |  | 16.5 |  |  |  |
| Tasar S et al., 2021 | Turkey | 98 | physicians | 2020 | 70.4 | 25-66 (30.5) | face to face | 94.9 |  |  |  |
| Tobaiqy M.A et al.,2023 | Saudi Arabia | 500 | parents | 2021 | 94.4 | 43.0±11.8 | face to face | 2.8 |  | 24.8 |  |
| Tolunay O et al., 2014 | Turkey | 228 | pediatrics, obstetrics and gynecology specialists | 2014 | 50.2 | 33.8±8.2 | self administered questionaire | 89.5 | 64.2 | 76.4 | 35.0 |
| Tonguc E et al., 2013 | Turkey | 945 | women | 2010 | 100 | 34.3±8.8 | self administered questionaire | 21.6 | 40.2 | 36.3 | 26 |
| Topan A et al., 2015 | Turkey | 396 | nurses | 2012 | 83.6 | 21-24 (83.3) | self administered questionaire |  | 56.8 |  |  |
| Topcu S et al., 2018 | Turkey | 147 | physicians | 2015 | 67 | 28.5 | self administered questionaire |  | 73 |  |  |
| Topçu S et al., 2017 | Turkey | 287 | communication and medicine students | 2015 | 47 | 23.1±1.5 |  | 48.3 | 55 |  |  |
| Tosun H. et al., 2022 | Turkey | 629 | female college students (faculty of health science) | 2018 | 100 | 19.5 ±1.6 | self administered questionaire | 19.4 |  |  |  |
| Tubas F et al., 2023 | Turkey | 200 | physicians nurses | 2020 | 70.5 | 27-37 (54) | face to face | 72 |  | 28.5 | 17 |
| Tunaman S.G et al., 2022 | Turkey | 275 | health services vocational school students | 2020 | 100 | 20.6±1.5 | self administered questionaire | 29.5 | 19.6 |  |  |
| Turan G et al., 2021 | Turkey | 836 | adults admitting to hospital | 2019 | 86.4 | 36.3 ±9.0 | self administered questionaire | 71.1 | 56.3 | 56.3 | 56.3 |
| Turhan E et al., 2017 | Turkey | 1087 | women and men who referred to the obstetrics and gynecology department | 2015 | 59 | 31.3±7.9 | self administered questionaire | 23.2 |  |  |  |
| Turker U.A et al., 2022 | Turkey | 380 | outpatient women | 2020 | 100 | <34 (80.1) | face to face | 17.3 |  |  |  |
| Turker-Aras U.A et al., 2023 | Turkey | 377 | mothers | 2022 | 100 | <40 (53.3) | face to face |  |  | 65 |  |
| Tursun S et al., 2022 | Turkey | 552 | parents | 2020 | 35.4 | 38.7±5.0 | face to face | 35.8 | 79.9 | 74.8 | 73.3 |
| Ulus B et al., 2017 | Turkey | 252 | mothers | 2014 | 100 | 28-47 (68.7) | face to face | 45.2 |  | 70.6 |  |
| Unutkan A et al., 2016 | Turkey | 468 | college students | 2014 | 100 | 21.1±2.0 | self administered questionaire | 56.4 | 31.4 |  |  |
| Uzuner A et al., 2018 | Turkey | 318 | adult | 2016 | 69.8 | 43,4±15,8 | face to face | 49.6 |  |  |  |
| Uzunlar O et al., 2013 | Turkey | 752 | nursing students and wommen | 2012 | 100 | 19.4±2.3 | self administered questionaire | 60.9 | 50.0 | 67.1 | 55.9 |
| Yagan Z et al.,2022 | Turkey | 250 | medical students | 2022 | 47.6 |  | online | 42 | 58.8 |  |  |
| Yaksi N et al., 2023 | Turkey | 339 | healthcare workers | 2022 | 74.9 | 36 (23-64) | online | 94.4 |  |  |  |
| Yalaki Z et al., 2016 | Turkey | 564 | high school students | 2015 | 58.2 | 17 [[16-19]] | self administered questionaire |  | 12.2 |  |  |
| Yarici F et al., 2023 | Cyprus | 1108 | adults | 2022 | 51.9 | 18-45 (100) | online | 36.6 |  |  |  |
| Yılmaz T et al., 2021 | Turkey | 533 | female students at Health Sciences | 2018 | 100 | 20.4±1.5 | face to face | 49.7 | 48.4 |  |  |
| Yigitalp G , 2019 | Turkey | 453 | nurses and midwiferies | 2018 | 66.4 | 34.4±8.6 | self administered questionaire | 48.8 | 15.9 |  |  |
| Yilmazel G et al., 2014 | Turkey | 463 | university students | 2013 | 76.9 | 20.7±2.4 |  | 48.6 | 15.2 |  |  |
| Yuksel B et al., 2014 | Turkey | 153 | doctors, nurses, and staff working at hospital | 2014 | 60 | 33.7±7.5 | self administered questionaire |  | 41.2 | 67 | 50 |
| Yurtsev E et al., 2013 | Turkey | 1062 | adolecent girls and mothers | 2010 | 100 | 26.9 | self administered questionaire |  | 40.5^3^ | 41.5^4^ |  |
| Zahid H.M et al., 2022 | Saudi Arabia | 1489 | adult women |  | 100 | 18-25 (51.4) | online | 12.6 |  |  |  |

**Supplementary table 3. Bias assessment of including studies**

| First author, year | 1.  Was the sample frame appropriate to address the target population? | 2. Were study participants sampled in an appropriate way? | 3. Was the sample size adequate? | 4.Were the study subjects and the setting described in detail? | 5.Was the data analysis conducted with sufficient coverage of the identified sample? | 6.Were valid methods used for the identification of the condition? | 7. Was the condition measured in a standard, reliable way for all participants? | 8. Was there appropriate statistical analysis? | 9.Was the response rate adequate, and if not, was the low response rate managed appropriately? | Total bias points |
| --- | --- | --- | --- | --- | --- | --- | --- | --- | --- | --- |
| Abou El-Ola M.J et al., 2018 | 1 | 0 | 1 | 1 | 0 | 1 | 1 | 1 | 0 | 6 |
| Abuduzike G et al., 2022 | 1 | 1 | 1 | 1 | (unclear) 0 | 1 | 1 | 1 | 1 | 8 |
| Acar O.N et al., 2019 | 1 | 1 | 1 | 1 | (unclear) 0 | 1 | 1 | 1 | 1 | 8 |
| Acoglu E.A et al., 2019 | 0 | 0 | 0 | 1 | (unclear) 0 | 1 | 1 | 1 | (unclear) 0 | 4 |
| Adiguzel F.I et al., 2016 | 0 | 0 | 1 | 1 | (unclear) 0 | 1 | 1 | 1 | (unclear) 0 | 5 |
| Aga S.S et al., 2022 | 1 | 0 | 1 | 1 | (unclear) 0 | 1 | 1 | 1 | (unclear) 0 | 6 |
| Akca G et al, 2022 | 0 | 1 | 1 | 1 | (unclear) 0 | 1 | 1 | 1 | 1 | 7 |
| Akkour K et al., 2021 | 0 | 0 | 1 | 1 | 0 | 1 | 1 | 1 | 1 | 6 |
| Akyuz A et al., 2011 | 0 | 0 | 1 | 1 | (unclear) 0 | 1 | 1 | 1 | (unclear) 0 | 5 |
| Al Darwish A.A et al., 2014 | 1 | (unclear) 0 | 0 | 0 | (unclear) 0 | 1 | 1 | 1 | (unclear) 0 | 4 |
| Al Raisi M et al., 2021 | 1 | 1 | 1 | 1 | 1 | 1 | 1 | 1 | 1 | 9 |
| Al-Nuaimi N.S et al, 2011 | 1 | 1 | 1 | 1 | (unclear) 0 | 1 | 1 | 1 | 1 | 8 |
| Al-Saadi A.N et al., 2021 | 1 | 1 | 1 | 1 | (unclear) 0 | 1 | 1 | 1 | 1 | 8 |
| Alawi S.A et al., 2023 | 1 | 0 | 1 | 0 | 0 | 1 | 1 | 1 | (unclear) 0 | 5 |
| Aldhafar A.S et al., 2016 | 0 | (unclear) 0 | 0 | 0 | (unclear) 0 | 1 | 1 | 1 | (unclear) 0 | 3 |
| Aldohaian A.I et al., 2019 | 1 | 1 | 1 | 1 | (unclear) 0 | 1 | 1 | 1 | (unclear) 0 | 7 |
| AlHarfi M.M.A al., 2019 | 1 | (unclear) 0 | 1 | 1 | (unclear) 0 | 1 | 1 | 1 | (unclear) 0 | 6 |
| Alhusayn K.O et al., 2022 | 0 | 0 | 1 | 1 | (unclear) 0 | 1 | 1 | 1 | (unclear) 0 | 5 |
| Ali M.D et al., 2022 | 1 | (unclear) 0 | 0 | 0 | 0 | 1 | 1 | 1 | (unclear) 0 | 4 |
| Alkalash S.H et al., 2022 | 1 | 0 | 1 | 1 | (unclear) 0 | 1 | 1 | 1 | (unclear) 0 | 6 |
| Alkan S et al.,2021 | 0 | 0 | 0 | 1 | (unclear) 0 | 1 | 1 | 1 | (unclear) 0 | 4 |
| Alkhaldi R.O et al., 2023 | 0 | 0 | 1 | 1 | (unclear) 0 | 1 | 1 | 1 | (unclear) 0 | 5 |
| Almaghlouth A.K et al., 2022 | 0 | 0 | 1 | 1 | (unclear) 0 | 1 | 1 | 1 | (unclear) 0 | 5 |
| Almazroua S et al., 2020 | 1 | 1 | 0 | 0 | 0 | 1 | 1 | 1 | (unclear) 0 | 5 |
| Almehmadi M.M et al., 2019 | 0 | 0 | 1 | 1 | (unclear) 0 | 1 | 1 | 1 | (unclear) 0 | 5 |
| Alnafisah R.A et al., 2019 | 1 | 0 | 1 | 1 | (unclear) 0 | 1 | 1 | 1 | (unclear) 0 | 6 |
| Alqarawi S.A et al., 2023 | 0 | 0 | 1 | 1 | (unclear) 0 | 1 | 1 | 1 | (unclear) 0 | 5 |
| Alsanafi M et al., 2023 | 0 | (unclear) 0 | 1 | 0 | (unclear) 0 | 1 | 1 | 1 | (unclear) 0 | 4 |
| Alshammari F et al., 2022 | 1 | 1 | 1 | 1 | 0 | 1 | 1 | 1 | (unclear) 0 | 7 |
| Alshammiri S.M, 2022 | (unclear) 0 | (unclear) 0 | 1 | 0 | (unclear) 0 | 1 | 1 | 1 | (unclear) 0 | 4 |
| Alshehri A.M et al., 2021 | 0 | (unclear) 0 | 1 | 1 | (unclear) 0 | 1 | 1 | 0 | (unclear) 0 | 4 |
| Alsous M.M et al., 2021 | 1 | (unclear) 0 | 1 | 1 | (unclear) 0 | 1 | 1 | 1 | (unclear) 0 | 6 |
| Anfinan N.M, 2019 | 1 | 1 | 1 | 1 | 0 | 1 | 1 | 1 | 0 | 7 |
| Ates S.A et al., 2020 | 0 | 0 | 0 | 1 | (unclear) 0 | 1 | 1 | 1 | (unclear) 0 | 4 |
| Aynacı G et al., 2019 | 0 | 0 | 1 | 0 | (unclear) 0 | 1 | 1 | 1 | (unclear) 0 | 4 |
| Azer S.A et al., 2022 | 0 | (unclear) 0 | 0 | 0 | (unclear) 0 | 1 | 1 | 1 | (unclear) 0 | 3 |
| Bal-Yılmaz H et al., 2018 | 1 | 1 | 1 | 1 | 0 | 1 | 1 | 1 | (unclear) 0 | 7 |
| Bantun F et al., 2022 | 0 | 0 | (unclear) 0 | 1 | (unclear) 0 | 1 | 1 | 1 | (unclear) 0 | 4 |
| Barhamain A.S et al., 2022 | 0 | (unclear) 0 | 1 | 1 | (unclear) 0 | 1 | 1 | 1 | (unclear) 0 | 5 |
| Basar F et al., 2019 | 0 | 1 | 1 | 1 | (unclear) 0 | 1 | 1 | 1 | 1 | 7 |
| Baser E et al., 2019 | 0 | 0 | 1 | 1 | (unclear) 0 | 1 | 1 | 1 | (unclear) 0 | 5 |
| Borlu A et al., 2016 | 1 | 1 | 1 | 1 | (unclear) 0 | 1 | 1 | 1 | (unclear) 0 | 7 |
| Bulbul S et al., 2013 | 1 | (unclear) 0 | 1 | 1 | (unclear) 0 | 1 | 1 | 1 | (unclear) 0 | 6 |
| Celik P et al., 2021 | 0 | (unclear) 0 | 1 | 1 | (unclear) 0 | 1 | 1 | 1 | (unclear) 0 | 5 |
| Cenk H et al., 2022 | 1 | 0 | 1 | 1 | (unclear) 0 | 1 | 1 | 1 | (unclear) 0 | 6 |
| Cetin O et al., 2014 | 1 | (unclear) 0 | 1 | 1 | (unclear) 0 | 1 | 1 | 1 | (unclear) 0 | 6 |
| Charalambous I et al., 2019 | 1 | 1 | 1 | 1 | 1 | 1 | 1 | 1 | (unclear) 0 | 8 |
| Christodoulou A et al., 2019 | 1 | 1 | 1 | 1 | (unclear) 0 | 1 | 1 | 1 | 1 | 8 |
| Cimke V.S et al., 2018 | 0 | 1 | 1 | 1 | (unclear) 0 | 1 | 1 | 1 | (unclear) 0 | 6 |
| Cinar I.O et al., 2019 | 1 | 1 | 1 | 1 | (unclear) 0 | 1 | 1 | 1 | 1 | 8 |
| Dany M et al. 2015 | 1 | 0 | 0 | 1 | (unclear) 0 | 1 | 1 | 1 | (unclear) 0 | 5 |
| Donmez S et al., 2019 | 1 | 1 | 1 | 1 | (unclear) 0 | 1 | 1 | 1 | (unclear) 0 | 7 |
| Dursun P et al., 2009 | 0 | (unclear) 0 | 1 | 1 | (unclear) 0 | 1 | 1 | 1 | 0 | 5 |
| Durusoy R et al., 2010 | 1 | 1 | 1 | 1 | (unclear) 0 | 1 | 1 | 1 | 1 | 8 |
| Elnashar M.A et al., 2022 | 0 | (unclear) 0 | 1 | 1 | (unclear) 0 | 1 | 1 | 1 | (unclear) 0 | 5 |
| Elshami M et al., 2022 | 1 | 0 | 1 | 1 | (unclear) 0 | 1 | 1 | 1 | (unclear) 0 | 6 |
| Erbaydar N et al., 2016 | 1 | 1 | 1 | 1 | 0 | 1 | 1 | 1 | (unclear) 0 | 7 |
| Erbiyik H.I et al., 2021 | 0 | 0 | 1 | 1 | (unclear) 0 | 1 | 1 | 1 | 1 | 6 |
| Erdogan M et al., 2021 | 1 | 1 | 1 | 1 | (unclear) 0 | 1 | 1 | 1 | (unclear) 0 | 7 |
| Farsi N.J et al., 2020 | 1 | 1 | 1 | 0 | 1 | 1 | 1 | 1 | 1 | 8 |
| Farsi N.J et al., 2021 | 1 | 1 | 1 | 1 | 0 | 1 | 1 | 1 | (unclear) 0 | 7 |
| Genc R.E et al., 2013 | 0 | 1 | 1 | 1 | (unclear) 0 | 1 | 1 | 1 | 0 | 6 |
| Gokmen-Karasu A.F et al., 2019 | 1 | 1 | 1 | 1 | (unclear) 0 | 1 | 1 | 1 | 1 | 8 |
| Gol I et al., 2016 | 0 | 1 | 0 | 1 | (unclear) 0 | 1 | 1 | 1 | (unclear) 0 | 5 |
| Gorkem U et al., 2015 | 1 | (unclear) 0 | 0 | 1 | (unclear) 0 | 1 | 1 | 1 | (unclear) 0 | 5 |
| Gunes O et al., 2023 | 0 | 0 | (unclear) 0 | 1 | (unclear) 0 | 1 | 1 | 1 | (unclear) 0 | 4 |
| Gursoy M.Y et al., 2023 | 0 | 1 | 1 | 1 | (unclear) 0 | 1 | 1 | 1 | 1 | 7 |
| Guder S et al.,2022 | 0 (unclear) | 0 | 1 | 1 | (unclear) 0 | 1 | 1 | 1 | (unclear) 0 | 5 |
| Guducu N et al., 2012 | 1 | 0 | 1 | 1 | (unclear) 0 | 1 | 1 | 1 | (unclear) 0 | 6 |
| Guvenc G et al., 2012 | 0 | 1 | 1 | 1 | (unclear) 0 | 1 | 1 | 1 | 1 | 7 |
| Hashemipour M.A et al., 2019 | 1 | (unclear) 0 | 0 | 0 | (unclear) 0 | 1 | 1 | 1 | (unclear) 0 | 4 |
| Hendaus M.A et al., 2021 | 0 | (unclear) 0 | 1 | 1 | 1 | 1 | 1 | 1 | 1 | 7 |
| Husain Y et al., 2019 | 1 | 1 | 1 | 1 | 1 | 1 | 1 | 1 | 1 | 9 |
| Hussain A.N et al., 2016 | 0 | 0 | 1 | 1 | (unclear) 0 | 1 | 1 | 1 | (unclear) 0 | 5 |
| Ilter E et al., 2010 | 0 | 0 | 1 | 1 | (unclear) 0 | 1 | 1 | 1 | (unclear) 0 | 5 |
| Jassim G et al., 2018 | 1 | 1 | 1 | 1 | (unclear) 0 | 1 | 1 | 1 | (unclear) 0 | 7 |
| Karaoglu S.A et al., 2022 | 0 | 0 | 0 | 1 | (unclear) 0 | 1 | 1 | 1 | (unclear) 0 | 4 |
| Kayi I et al., 2020 | 1 | (unclear) 0 | 1 | 1 | (unclear) 0 | 1 | 1 | 1 | (unclear) 0 | 6 |
| Kazanci F et.al., 2022 | 1 | (unclear) 0 | (unclear) 0 | 0 | (unclear) 0 | 1 | 1 | 1 | (unclear) 0 | 4 |
| Keser G et al., 2020 | 1 | 0 | (unclear) 0 | 1 | (unclear) 0 | 1 | 1 | 1 | (unclear) 0 | 5 |
| Keten H.S, et al., 2021 | 1 | 0 | 1 | 1 | (unclear) 0 | 1 | 1 | 1 | (unclear) 0 | 6 |
| Khoury J.E et al., 2023 | 0 | 0 | 1 | 1 | (unclear) 0 | 1 | 1 | 1 | (unclear) 0 | 5 |
| Kilicaslan O et al., 2021 | 0 | 1 | 1 | 1 | 1 | 1 | 1 | 1 | 1 | 8 |
| Kiskac N et al., 2021 | 0 | 1 | 1 | 1 | (unclear) 0 | 1 | 1 | 1 | (unclear) 0 | 6 |
| Kizmaz M et al., 2022 | 0 | 0 | 1 | 1 | (unclear) 0 | 1 | 1 | 1 | (unclear) 0 | 5 |
| Kilic A et al., 2012 | 1 | 0 | 1 | 1 | (unclear) 0 | 1 | 1 | 1 | 1 | 7 |
| Koc Z et.al., 2015 | 1 | 1 | 1 | 1 | (unclear) 0 | 1 | 1 | 1 | 1 | 8 |
| Koc Z, 2014 | 0 | 1 | 1 | 1 | (unclear) 0 | 1 | 1 | 1 | 1 | 7 |
| Kose D et al., 2014 | 0 | (unclear) 0 | 1 | 1 | (unclear) 0 | 1 | 1 | 1 | (unclear) 0 | 5 |
| Kurtipek G.S et al., 2016 | 0 | 1 | 1 | 1 | (unclear) 0 | 1 | 1 | 1 | (unclear) 0 | 6 |
| Kurtoğlu E. et. Al. 2013 | 0 | 0 | 0 | 1 | (unclear) 0 | 1 | 1 | 1 | (unclear) 0 | 4 |
| Lataifeh I et al., 2016 | 1 | 0 | 1 | 0 | (unclear) 0 | 1 | 1 | 1 | 1 | 6 |
| Levent C et al., 2021 | 0 | (unclear) 0 | 1 | 1 | (unclear) 0 | 1 | 1 | 1 | (unclear) 0 | 5 |
| Lingam A.S et al., 2022 | 0 | (unclear) 0 | 1 | 0 | (unclear) 0 | 1 | 1 | 1 | 0 | 4 |
| Mahomed, M.F et al., 2020 | 0 | 0 | 1 | 1 | (unclear) 0 | 1 | 1 | 1 | (unclear) 0 | 5 |
| Mete B et al., 2023 | 1 | 1 | 1 | 1 | (unclear) 0 | 1 | 1 | 1 | (unclear) 0 | 7 |
| Mojahed S. et al., 2013 | 1 | 0 | 1 | 0 | (unclear) 0 | 1 | 1 | 1 | (unclear) 0 | 5 |
| Moosa K et al., 2014 | 1 | 1 | 1 | 1 | (unclear) 0 | 1 | 1 | 1 | (unclear) 0 | 7 |
| Naki M.M et al., 2016 | 0 | (unclear) 0 | 1 | 1 | (unclear) 0 | 1 | 1 | 1 | (unclear) 0 | 5 |
| Natan M.B et al., 2011 | 0 | 0 | 0 | 1 | (unclear) 0 | 1 | 1 | 1 | (unclear) 0 | 4 |
| Onan A et al., 2009 | 0 | 0 | 0 | 1 | (unclear) 0 | 1 | 1 | 1 | (unclear) 0 | 4 |
| Onder O. et al., 2015 | (unclear) 0 | 0 | 1 | 1 | (unclear) 0 | 1 | 1 | 1 | (unclear) 0 | 5 |
| Ortashi O et al., 2012 | 1 | 1 | 1 | 0 | (unclear) 0 | 1 | 1 | 1 | (unclear) 0 | 6 |
| Ortashi O et al., 2013 | 1 | 1 | 1 | 0 | (unclear) 0 | 1 | 1 | 1 | 1 | 7 |
| Ortashi O et al., 2014 | 0 | 0 | 1 | 1 | (unclear) 0 | 1 | 1 | 1 | 1 | 6 |
| Oz M et al., 2016 | 0 | 0 | 1 | 1 | 0 | 1 | 1 | 1 | (unclear) 0 | 5 |
| Ozcam H et al., 2014 | 0 | 0 | 0 | 1 | (unclear) 0 | 1 | 1 | 1 | (unclear) 0 | 4 |
| Ozdede M et al., 2020 | 0 | 0 | 0 | 1 | (unclear) 0 | 1 | 1 | 1 | (unclear) 0 | 4 |
| Ozsurekci Y et al., 2013 | 0 | (unclear) 0 | (unclear) 0 | 1 | (unclear) 0 | 1 | 1 | 1 | (unclear) 0 | 4 |
| Ozyer S et al., 2013 | 0 | 1 | 1 | 1 | (unclear) 0 | 1 | 1 | 1 | (unclear) 0 | 6 |
| Parlak E et al., 2021 | 0 | 0 | 1 | 1 | (unclear) 0 | 1 | 1 | 1 | (unclear) 0 | 5 |
| Purut Y.E et al., 2020 | 0 | 1 | 0 | 1 | (unclear) 0 | 1 | 1 | 1 | (unclear) 0 | 5 |
| Rathfisch G et al., 2015 | 0 | (unclear) 0 | 1 | 0 | (unclear) 0 | 1 | 1 | 1 | (unclear) 0 | 4 |
| Revanli R.A et al., 2016 | 0 | 0 | 1 | 1 | (unclear) 0 | 1 | 1 | 1 | (unclear) 0 | 5 |
| Sabr Y et al., 2021 | 1 | (unclear) 0 | 1 | 1 | (unclear) 0 | 1 | 1 | 1 | (unclear) 0 | 6 |
| Sahin H.G et al., 2017 | 0 | (unclear) 0 | 1 | 1 | (unclear) 0 | 1 | 1 | 1 | (unclear) 0 | 5 |
| Sahin H.O et al., 2020 | 1 | 1 | 1 | 1 | 0 | 1 | 1 | 1 | (unclear) 0 | 7 |
| Sahin R.A et al., 2022 | 1 | 1 | 1 | 1 | (unclear) 0 | 1 | 1 | 1 | 1 | 8 |
| Sait K.H, 2009 | 0 | 0 | 1 | 1 | (unclear) 0 | 1 | 1 | 1 | 1 | 6 |
| Sallam M et al., 2021 | 1 | 1 | 1 | 1 | (unclear) 0 | 1 | 1 | 1 | (unclear) 0 | 7 |
| Sallam M et al., 2022 | 1 | 1 | 1 | 1 | 0 | 1 | 1 | 1 | (unclear) 0 | 7 |
| Saqer A et al., 2017 | 0 | 0 | 1 | 0 | (unclear) 0 | 1 | 1 | 1 | (unclear) 0 | 4 |
| Sargazi N et al., 2021 | (unclear) 0 | (unclear) 0 | (unclear) 0 | 0 | 0 | 1 | 1 | 1 | (unclear) 0 | 3 |
| Senol V et al., 2011 | 1 | 1 | 1 | 1 | 1 | 1 | 1 | 1 | 1 | 9 |
| Seven M et al., 2015 | 0 | 1 | 1 | 1 | (unclear) 0 | 1 | 1 | 1 | 1 | 7 |
| Shdefat S.A et al., 2022 | 0 | 0 | 1 | 0 | (unclear) 0 | 1 | 1 | 1 | (unclear) 0 | 4 |
| Shibli R et al., 2019 | 1 | 1 | 1 | 1 | (unclear) 0 | 1 | 1 | 1 | 1 | 8 |
| Sundaram M.K et al., 2021 | 0 | 0 | 1 | 1 | (unclear) 0 | 1 | 1 | 1 | (unclear) 0 | 5 |
| Tas B et al., 2016 | 0 | (unclear) 0 | 1 | 1 | (unclear) 0 | 1 | 1 | 1 | (unclear) 0 | 5 |
| Tas F et al., 2010 | 0 | 1 | 1 | 1 | 0 | 1 | 1 | 1 | (unclear) 0 | 6 |
| Tasar S et al., 2021 | 1 | 1 | 0 | 1 | (unclear) 0 | 1 | 1 | 1 | 1 | 7 |
| Tobaiqy M.A et al.,2023 | 0 | 0 | 1 | 1 | (unclear) 0 | 1 | 1 | 1 | 1 | 6 |
| Tolunay O et al., 2014 | (unclear) 0 | 0 | 1 | 1 | (unclear) 0 | 1 | 1 | 1 | (unclear) 0 | 5 |
| Tonguc E et al., 2013 | 1 | 1 | 1 | 1 | (unclear) 0 | 1 | 1 | 1 | 1 | 8 |
| Topan A et al., 2015 | 1 | 1 | 1 | 0 | (unclear) 0 | 1 | 1 | 1 | (unclear) 0 | 6 |
| Topcu S et al., 2018 | 0 | 1 | 0 | 1 | (unclear) 0 | 1 | 1 | 1 | (unclear) 0 | 5 |
| Topçu S et al., 2017 | 0 | 0 | 1 | 1 | (unclear) 0 | 1 | 1 | 1 | (unclear) 0 | 5 |
| Tosun H. et al., 2022 | 0 | (unclear) 0 | 1 | 1 | 1 | 1 | 1 | 1 | 1 | 7 |
| Tubas F et al., 2023 | 0 | 0 | (unclear) 0 | 0 | 0 | 1 | 1 | 1 | (unclear) 0 | 3 |
| Tunaman S.G et al., 2022 | 0 | 1 | 0 | 1 | (unclear) 0 | 1 | 1 | 1 | (unclear) 0 | 5 |
| Turan G et al., 2021 | 0 | (unclear) 0 | 1 | 1 | (unclear) 0 | 1 | 1 | 1 | (unclear) 0 | 5 |
| Turhan E et al., 2017 | 0 | 0 | 1 | 1 | 1 | 1 | 1 | 1 | 1 | 7 |
| Turker U.A et al., 2022 | 0 | (unclear) 0 | 1 | 1 | (unclear) 0 | 1 | 1 | 1 | (unclear) 0 | 5 |
| Turker-Aras U.A et al., 2023 | 1 | (unclear) 0 | 1 | 0 | (unclear) 0 | 1 | 1 | 1 | (unclear) 0 | 5 |
| Tursun S et al., 2022 | 0 | 0 | 1 | 1 | (unclear) 0 | 1 | 1 | 1 | (unclear) 0 | 5 |
| Ulus B et al., 2017 | 0 | (unclear) 0 | (unclear) 0 | 1 | (unclear) 0 | 1 | 1 | 1 | (unclear) 0 | 4 |
| Unutkan A et al., 2016 | 0 | 0 | 1 | 1 | (unclear) 0 | 1 | 1 | 1 | 0 | 5 |
| Uzuner A et al., 2018 | 1 | 0 | 1 | 1 | (unclear) 0 | 1 | 1 | 1 | (unclear) 0 | 6 |
| Uzunlar O et al., 2013 | 1 | 0 | 1 | 1 | (unclear) 0 | 1 | 1 | 1 | 1 | 7 |
| Yagan Z et al.,2022 | 0 | 1 | 1 | 1 | (unclear) 0 | 1 | 1 | 1 | (unclear) 0 | 6 |
| Yaksi N et al., 2023 | 0 | 0 | 1 | 1 | (unclear) 0 | 1 | 1 | 1 | (unclear) 0 | 5 |
| Yalaki Z et al., 2016 | 0 | (unclear) 0 | 1 | 0 | (unclear) 0 | 1 | 1 | 1 | (unclear) 0 | 4 |
| Yarici F et al., 2023 | 0 | 0 | 1 | 0 | (unclear) 0 | 1 | 1 | 1 | (unclear) 0 | 4 |
| Yılmaz T et al., 2021 | 1 | (unclear) 0 | 1 | 1 | (unclear) 0 | 1 | 1 | 1 | (unclear) 0 | 6 |
| Yigitalp G , 2019 | 1 | 0 | 1 | 1 | (unclear) 0 | 1 | 1 | 1 | (unclear) 0 | 6 |
| Yilmazel G et al., 2014 | 1 | 1 | 1 | 1 | (unclear) 0 | 1 | 1 | 1 | (unclear) 0 | 7 |
| Yuksel B et al., 2014 | 0 | (unclear) 0 | 0 | 0 | (unclear) 0 | 1 | 1 | 1 | (unclear) 0 | 3 |
| Yurtsev E et al., 2013 | 0 | 1 | 1 | 1 | (unclear) 0 | 1 | 1 | 1 | (unclear) 0 | 6 |
| Zahid H.M et al., 2022 | 1 | 0 | 1 | 1 | (unclear) 0 | 1 | 1 | 1 | (unclear) 0 | 6 |


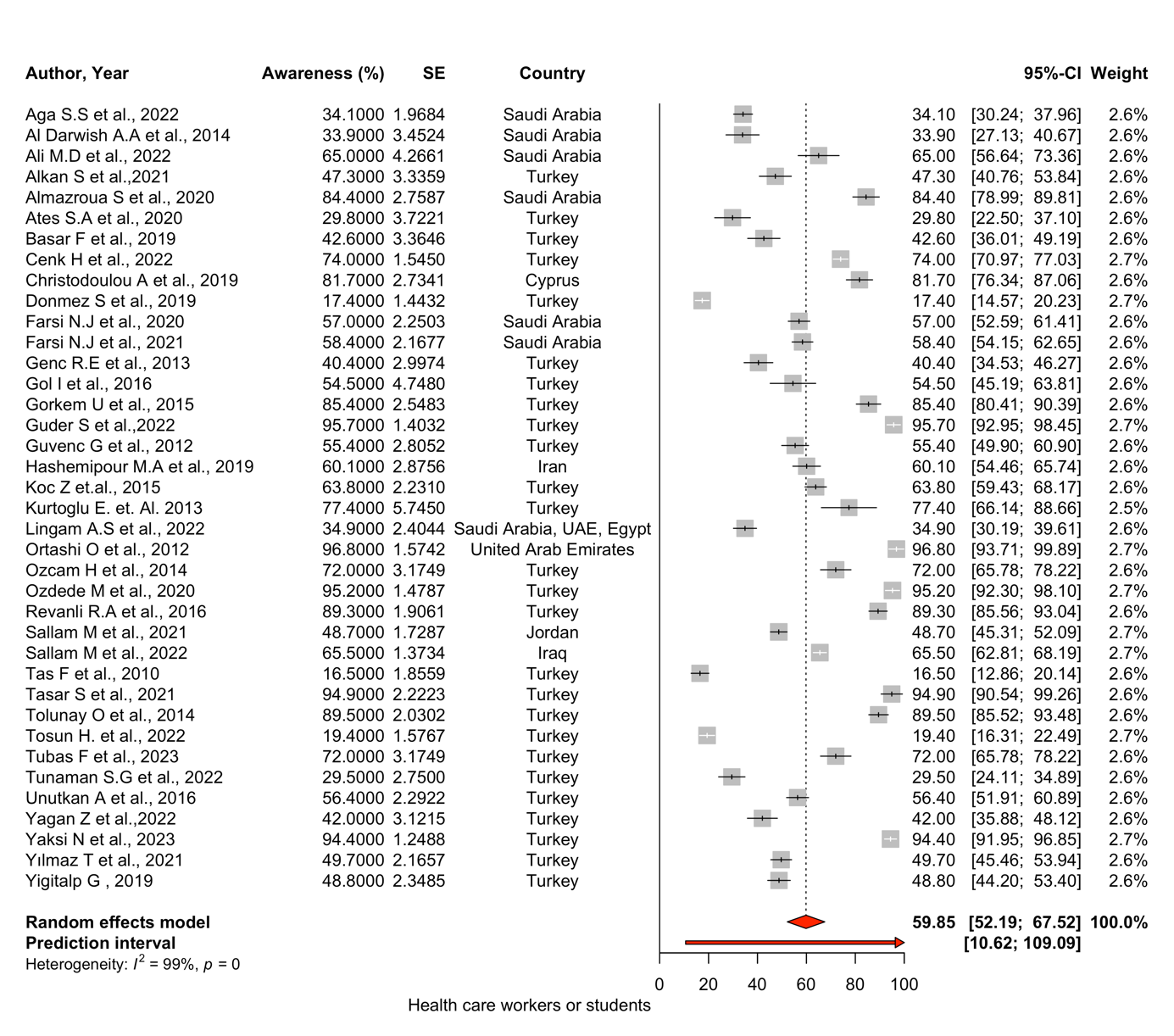


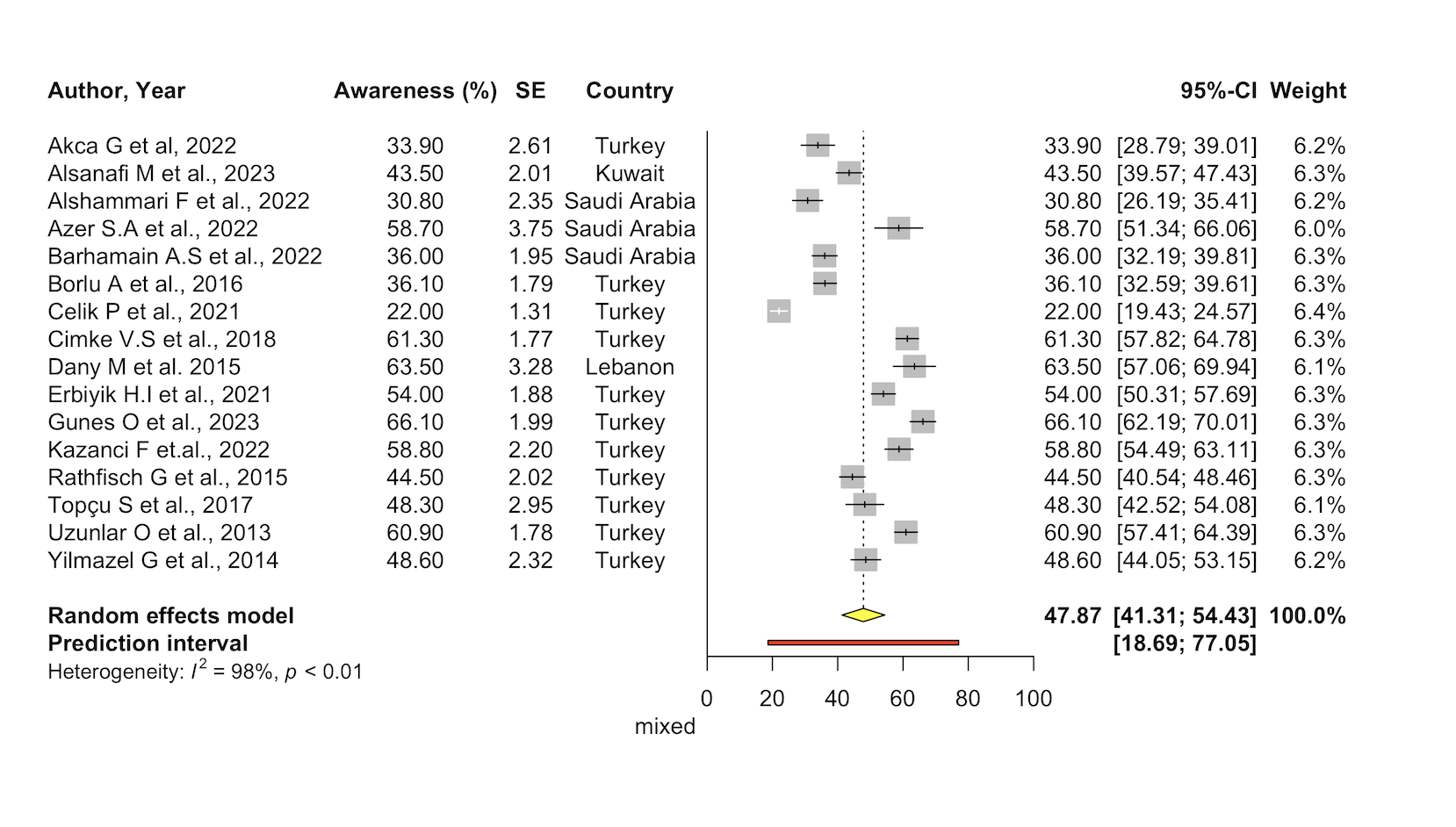


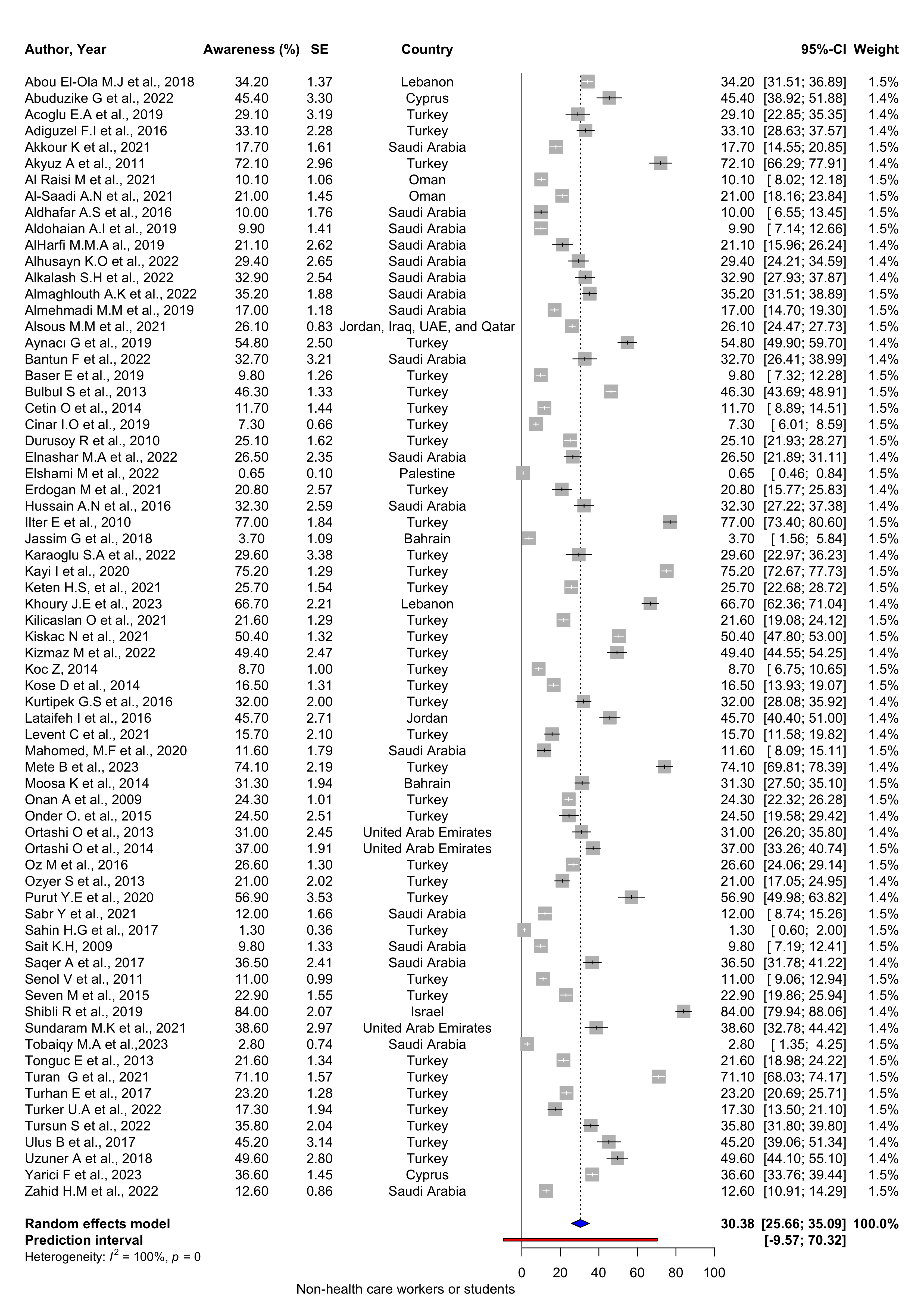


Healthcare workers or students refers to studies where at least 90% of the study population consisted of healthcare workers or students. Mixed refers to studies where the proportion of healthcare workers or students is between 10% and 90%. Non-healthcare workers or students refers to studies where 10% or less of the study population consisted of healthcare workers or students.

**Supplementary Figure 1. The forest plot for HPV vaccine awareness categorized based on the inclusion of healthcare workers**

**
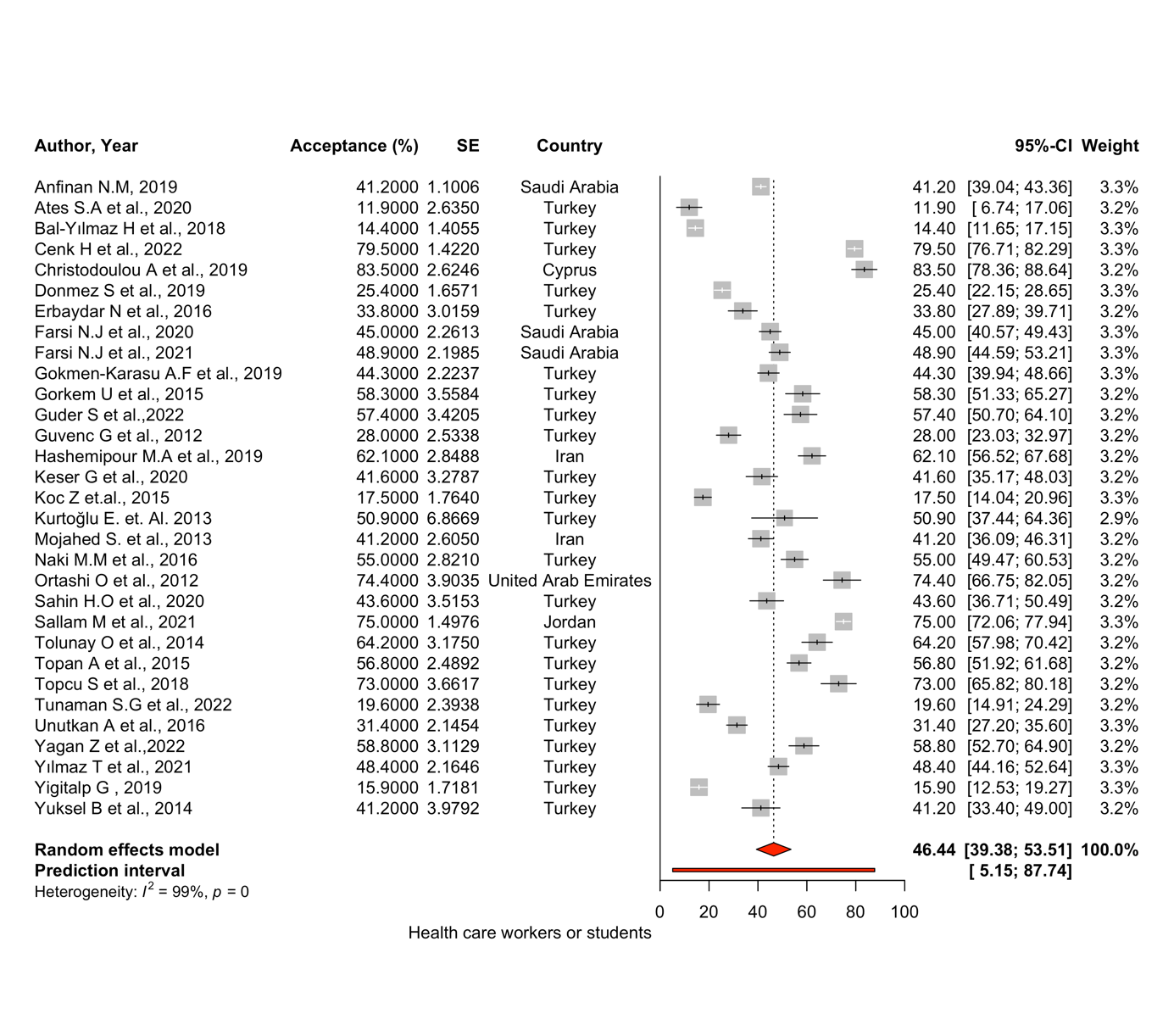
**

**
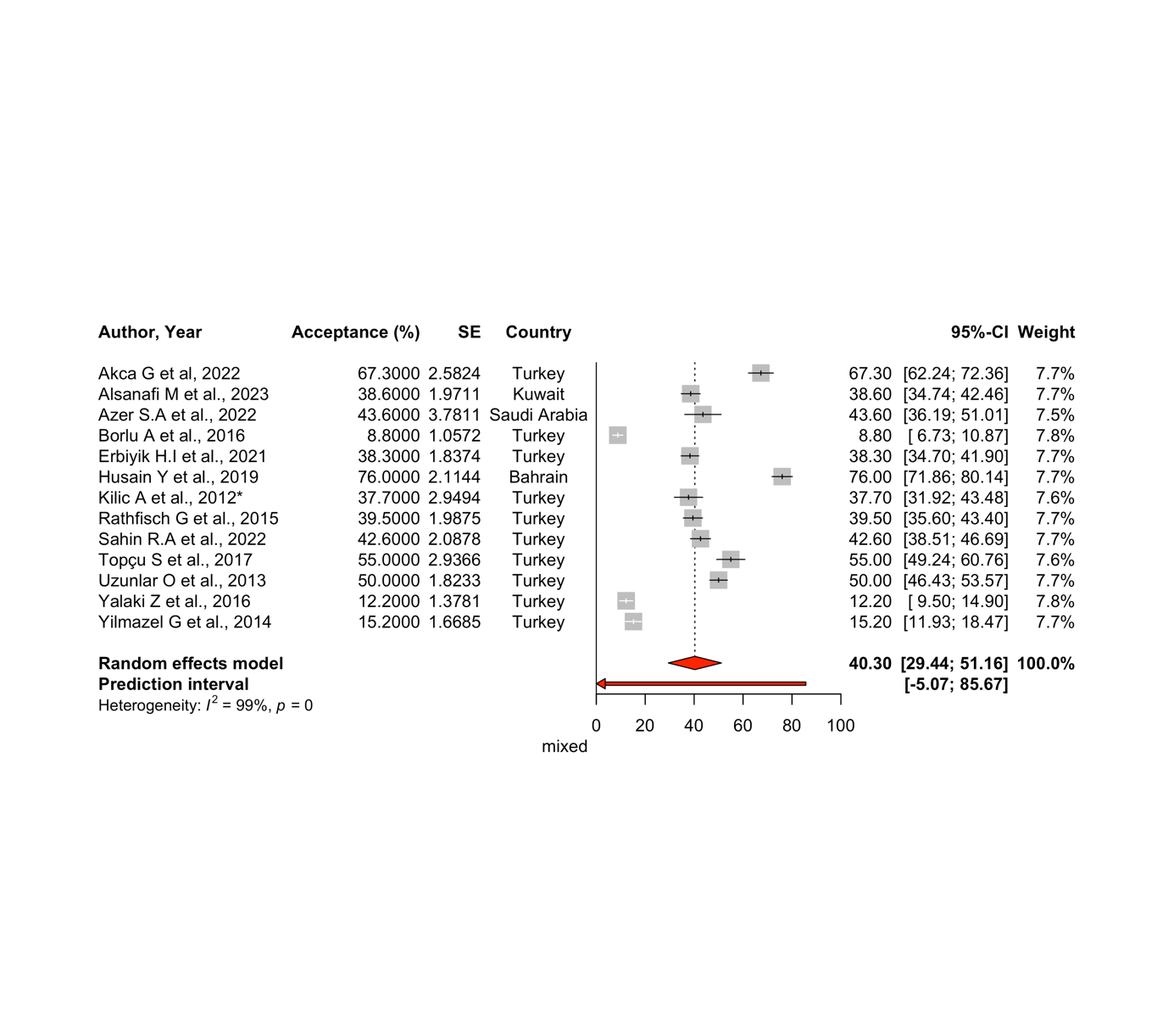
**

**
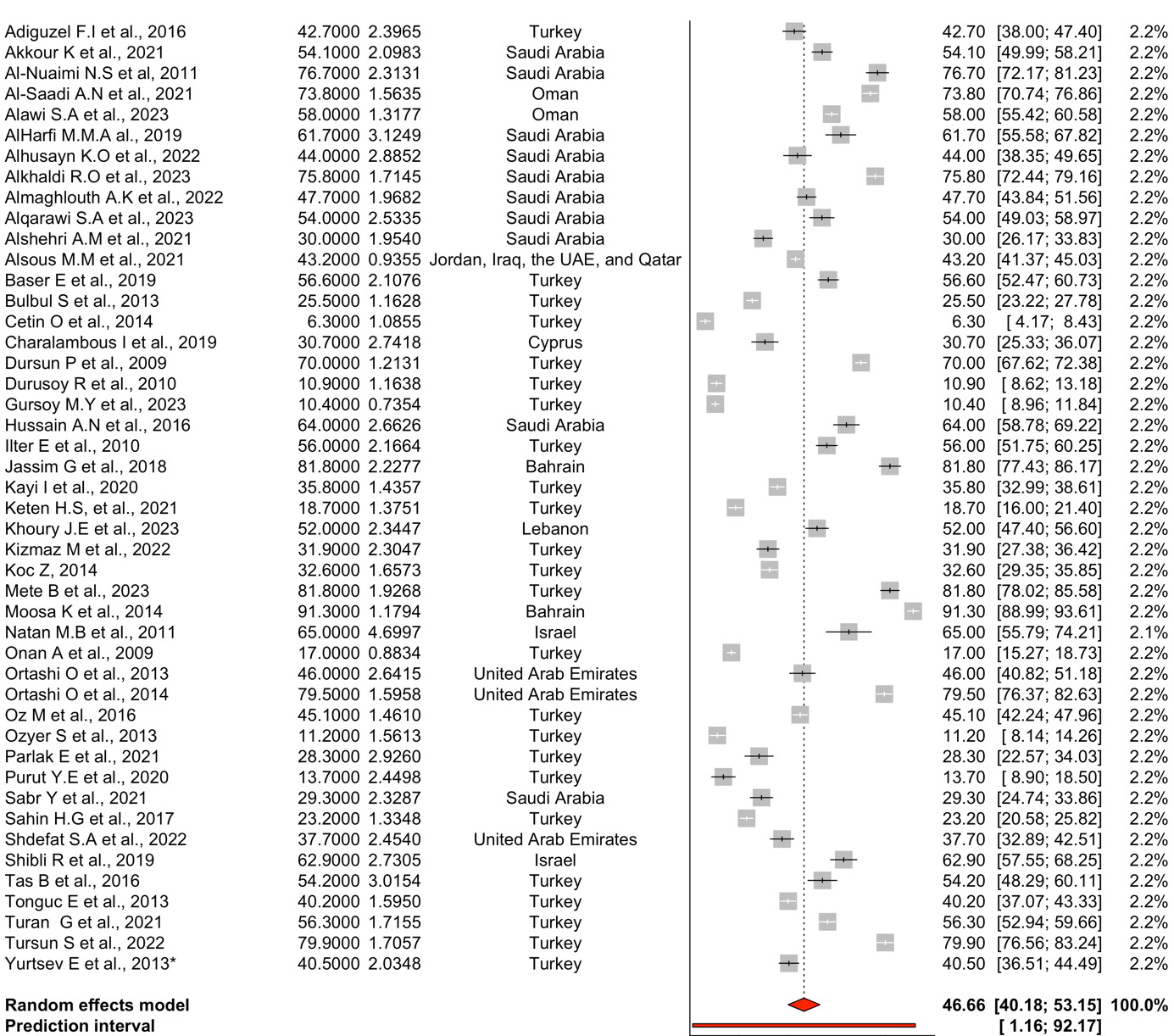
**

Non-health care workers or students

Healthcare workers or students refers to studies where at least 90% of the study population consisted of healthcare workers or students. Mixed refers to studies where the proportion of healthcare workers or students is between 10% and 90%. Non-healthcare workers or students refers to studies where 10% or less of the study population consisted of healthcare workers or students.

**Supplementary Figure 2. The forest plot for HPV vaccine acceptance categorized based on the inclusion of healthcare workers or**
